# Genome sequence of *Jatropha curcas* L., a non‐edible biodiesel plant, provides a resource to improve seed‐related traits

**DOI:** 10.1111/pbi.12995

**Published:** 2018-09-11

**Authors:** Jungmin Ha, Sangrea Shim, Taeyoung Lee, Yang J. Kang, Won J. Hwang, Haneul Jeong, Kularb Laosatit, Jayern Lee, Sue K. Kim, Dani Satyawan, Puji Lestari, Min Y. Yoon, Moon Y. Kim, Annapurna Chitikineni, Patcharin Tanya, Prakit Somta, Peerasak Srinives, Rajeev K. Varshney, Suk‐Ha Lee

**Affiliations:** ^1^ Department of Plant Science and Research Institute of Agriculture and Life Sciences Seoul National University Seoul Korea; ^2^ Plant Genomics and Breeding Institute Seoul National University Seoul Korea; ^3^ Division of Applied Life Science (BK21 plus program) Department Gyeongsang National University PMBBRC Jinju‐si Korea; ^4^ Division of Life Science Department Gyeongsang National University Jinju‐si Korea; ^5^ CJ Food R&D Suwon Korea; ^6^ Department of Agronomy Faculty of Agriculture at Kamphaeng Saen Kasetsart University Nakhon Pathom Thailand; ^7^ Department of Chemistry College of Natural Science Dankook University Cheonan South Korea; ^8^ Indonesian Center for Agricultural Biotechnology and Genetic Resources Research and Development (ICABIOGRAD‐IAARD) Bogor Indonesia; ^9^ Center of Excellence in Genomics & Systems Biology International Crops Research Institute for the Semi‐Arid Tropics (ICRISAT) Hyderabad Telangana State India

**Keywords:** oil synthesis, phorbol ester, biodiesel, seed cake, energy production, phylogenetic analysis

## Abstract

*Jatropha curcas* (physic nut), a non‐edible oilseed crop, represents one of the most promising alternative energy sources due to its high seed oil content, rapid growth and adaptability to various environments. We report ~339 Mbp draft whole genome sequence of *J. curcas* var. Chai Nat using both the PacBio and Illumina sequencing platforms. We identified and categorized differentially expressed genes related to biosynthesis of lipid and toxic compound among four stages of seed development. Triacylglycerol (TAG), the major component of seed storage oil, is mainly synthesized by phospholipid:diacylglycerol acyltransferase in Jatropha, and continuous high expression of homologs of oleosin over seed development contributes to accumulation of high level of oil in kernels by preventing the breakdown of TAG. A physical cluster of genes for diterpenoid biosynthetic enzymes, including casbene synthases highly responsible for a toxic compound, phorbol ester, in seed cake, was syntenically highly conserved between Jatropha and castor bean. Transcriptomic analysis of female and male flowers revealed the up‐regulation of a dozen family of TFs in female flower. Additionally, we constructed a robust species tree enabling estimation of divergence times among nine *Jatropha* species and five commercial crops in Malpighiales order. Our results will help researchers and breeders increase energy efficiency of this important oil seed crop by improving yield and oil content, and eliminating toxic compound in seed cake for animal feed.

## Introduction

Sustainable biofuel has been receiving increasing attention as an alternative energy source to fossil fuels due to increasing greenhouse gas emissions and energy consumption. Among several biofuel plants, *Jatropha curcas* (physic nut), a non‐edible oilseed crop, is one of the most promising biofuel feedstocks because it has high seed oil content, drought tolerance, rapid growth and adaptability to a wide range of climatic and soil conditions (Kumar and Sharma, 2008). Physic nut is a perennial, monoecious tree or shrub belonging to the Euphorbiaceae family, which includes many economically important crops such as rubber tree (*Hevea brasiliensis*), cassava (*Manihot esculenta*) and castor bean (*Ricinus communis*). It has very small chromosomes (1.24–1.71 μm) with 2n = 2*x* = 22 and a relatively small genome size (*C* = 416 Mb) (Carvalho *et al*., 2008). Physic nut is native to Central America and has been grown commercially and/or non‐commercially in smallholder farms and plantations in tropical and sub‐tropical Asia and Africa (van Eijck *et al*., 2014; Iiyama *et al*., 2013; Kalam *et al*., 2012; Silitonga *et al*., 2011). Even before Jatropha was promoted as a bioenergy crop, it was often grown as fencing, hedging or a windbreak around homesteads, and it has since become useful for generating cash for smallholder farmers (van Eijck *et al*., 2014). The roles played by physic nut in poverty reduction in rural areas and energy generation as biodiesel have given it widespread acceptance in developing countries, in contrast with oil palm which is mainly grown for commercial farming (Kalinda *et al*., 2015; von Maltitz *et al*., 2014). However, many farmers have given up on growing Jatropha for biodiesel production because of its unexpectedly low yields due to a lack of elite cultivars and a poor understanding of the basic agronomy of Jatropha.

Jatropha is less domesticated, and has much potential to be improved through breeding programs (Iiyama *et al*., 2013; Mas'ud, 2016). The genetic improvement of Jatropha should focus on obtaining high seed yield with high oil content, more female flowers, and low phorbol ester (PE) content, which would make seed cake less toxic. Enhancing our knowledge of genetic variation in germplasm collections is crucial for successful genetic improvement. The oil content and 100‐seed weight of Jatropha vary from 28 to 39% and from 44 to 77 g, respectively, depending on genotypes, which are significantly correlated (Kaushik *et al*., 2007; Wani *et al*., 2006). As a monoecious plant, the male‐to‐female flower ratio is significantly correlated to seed yield (Wijaya *et al*., 2009). The presence of PE makes Jatropha undervalued as a potential biofuel feedstock. The amount of energy obtained from the resulting seed cake after oil extraction is similar to that of whole Jatropha seeds (Jongschaap *et al*., 2009). A non‐toxic variety of Jatropha is found in Mexico, and the Agricultural Research Trust in Zimbabwe has developed a non‐toxic variety of *J. curcas*, which would make the seed cake usable for animal consumption without extra cost for detoxification (Makkar *et al*., 1998; Prusty *et al*., 2008). Transgenic Jatropha with less PE has recently been generated via RNAi (Li *et al*., 2015). However, although such agronomical and molecular studies have been conducted on Jatropha, its potential as a source of biofuel and animal feedstock has not yet been realized.

Jatropha seeds comprise up to 40% oil in whole seeds and 58% in kernels. The main constituents of Jatropha seed oil, oleic, linoleic and palmitic acid, make the oil an efficient substitute for standard diesel oil (Ginwal *et al*., 2004; Gübitz *et al*., 1999). Defatted Jatropha kernels obtained from seed cake, a byproduct of Jatropha oil production, comprise 56–63% protein, which is higher than the protein content in commercial soybean meal (46.5%) (Makkar *et al*., 1998). In contrast to first‐generation biofuel crops such as corn, soybean and rapeseed, physic nut does not threaten food security as it is a non‐edible oil seed crop. In addition, weak crassulacean acid metabolism occurs in the succulent stems of physic nut. This characteristic provides physic nut with drought tolerance and makes it well‐adapted to arid lands (Maes *et al*., 2009), thereby preventing competition for arable lands used for food production.

To improve crop quality and yield in members of the Euphorbiaceae family to provide industrial raw materials and to increase the food supply, a high‐quality reference genome sequence and comprehensive analysis of genomic and transcriptomic data across the species are required. Several economically valuable crops in the Euphorbiaceae family have been sequenced. The castor bean genome sequence is 350.6 Mb in size (110% of the estimated genome size of ~320 Mbp), with a scaffold N50 length of 496.5 kb (Chan *et al*., 2010). The rubber tree genome sequence comprises 1.34 Gbp (93.8% of the estimated genome size, ~1.5 Gb), with a scaffold N50 length of 1.3 Mb (Tang *et al*., 2016). The cassava genome sequence is 432 Mbp in size (58.2% of the estimated genome size of ~740 Mbp), with a scaffold N50 length of 43 kbp (Wang *et al*., 2014b). Finally, the previously reported genome sequence of physic nut is 320.5 Mb in size, with a scaffold N50 length of 746 kbp; 81.7% of this assembly was anchored to a linkage map containing 1208 markers (Wu *et al*., 2015).

In the present study, we constructed an improved, high‐quality genome assembly of *J. curcas* var. Chai Nat (CN) on a chromosomal scale using both the PacBio and Illumina sequencing platforms. Comprehensive transcriptomic analysis on nine different tissues of *J. curcas* and nine *Jatropha* species was performed to explore the biosynthesis of lipids and toxic compounds and speciation in the genus *Jatropha*. Phylogenetic and comparative analyses of six species in the order Malpighiales shed light on the evolution of economically important crops in the Euphorbiaceae family. The primary genome information for physic nut obtained in this study will facilitate genomics research in the Euphorbiaceae family and accelerate Jatropha breeding programs by providing a platform for the discovery of genes affecting oil yield, oil quality and toxic compounds.

## Results

### Genome assembly and genetic map construction

We sequenced and performed *de novo* assembly of the genome of *Jatropha curcas* var. CN using PacBio long reads and Illumina short reads (Tables 1 and S1, Figure S1, Data S1) (Allen *et al*., 2006). A total of 32.6 Gbp (78× coverage of the estimated genome size) generated by PacBio were assembled into 1736 contigs (Table 1). The contigs were assembled into 917 scaffolds with an N50 of 1.5 Mbp using 133.5 Gbp of Illumina mate pair reads (Figure S2). To construct a genetic map, we genotyped 108 F_2_ lines derived from a cross between *J. curcas* CN and *J. curcas* M10 by genotyping‐by‐sequencing (GBS) (Elshire *et al*., 2011), with an average mapping depth of 15× (Data S2) (Li *et al*., 2009). We identified 1592 markers, 1186 of which were used to construct 11 linkage groups, representing 738.1 cM (Table S2). To anchor the scaffolds onto pseudochromosomes using ALLMAPS which uses a combination of multiple maps to improve the accuracy of the resulting chromosomal assemblies, we constructed a secondary genetic map consisting of 864 markers (Table S2; Tang *et al*., 2015). A total of 116 scaffolds spanning 204 Mbp were anchored into 11 superscaffolds using 1770 unique markers (Figure S3). If only superscaffolds larger than 2 kbp are considered, the assembly spans 339.4 Mbp (82% of the estimated genome size), with an N50 of 15.4 Mbp containing 0.24% of Ns (Table 1; Carvalho *et al*., 2008). Core Eukaryotic Genes Mapping Approach (CEGMA) analysis showed that 85.9% of core eukaryotic gene sequences were complete (97.2% were partial), and Benchmarking Universal Single‐Copy Orthologs (BUSCO) showed that 82.5% of embryophyta gene sequences were complete (87.8% were partial), in our assembly (Table S3; Parra *et al*., 2007; Simão *et al*., 2015). The level of heterozygosity in *J. curcas* CN was measured by mapping Illumina paired end reads against the assembly. We identified 0.59 single nucleotide variations and 0.06 insertions and deletions per 1 kbp (Table S4). The level of heterozygosity was relatively low compared with poplar (2.6/kb) and cassava (3.4/kb) in Malpighiales, which is consistent with the previous finding that fruiting by self‐pollination in *J. curcas* ranges from 72.2% to 93.2% (Kaur *et al*., 2011; Luo *et al*., 2007; Tuskan *et al*., 2006; Wang *et al*., 2014b).

**Table 1:** Statistics for *J. curcas* CN genome assembly and gene annotation

	Contig	Scaffold	Superscaffold	Superscaffold >2k
Total bases	338 314 442	339 339 362	339 501 388	339 366 681
Total number	1736	917	812	681
Maximum length	5 796 004	11 371 878	28 673 349	28 673 349
Minimum length	145	145	145	2031
N50 length	1 013 964	1 476 473	15 395 338	15 395 338
Mean length	194 881.59	370 054.00	418 105	498 336
GC content (%)	34.96	34.85	34.88	34.88
% of Ns	0.00	0.30	0.24	0.24
Avg. length of breakage (>25 Ns) between contigs	–	1690	1611	1611
Number of gene models	–	–	27 619	–
Number of transcripts	–	–	27 680	–

Assembly statistics were collected from four stages of genome assemblies. The scaffolds shorter than 2 kbp were filtered out from gap‐filled superscaffolds for the final statistics. Gaps between scaffolds in superscaffolds were filled with 100 Ns.

### Genome annotation

We further confirmed the quality and coverage of the assembly using transcript sequences. Of the 1.4 million transcripts from five different tissues with three replications, 95% transcripts were properly mapped to the assembly (Data S1, Table S5). Using the transcriptome data, consisting of non‐redundant 169 670 transcripts and the *ab initio* gene prediction, 27 619 gene models were predicted, which is fewer than the number of genes predicted from other genomes with similar estimated genome sizes, such as poplar (45 555 gene models/410 Mbp; Tuskan *et al*., 2006), rice (37 544 gene models/389 Mbp; Project, 2005) and castor bean (31 237 gene models/320 Mbp) (Table S6; Chan *et al*., 2010). We identified 59.35% of the genome assembly as repeat sequences, of which long‐terminal repeat retrotransposons (LTR‐RTs), mainly *Gypsy* (28.54%) and *Copia* (7.98%), were the most abundant (Table S7).

**Figure 1:**
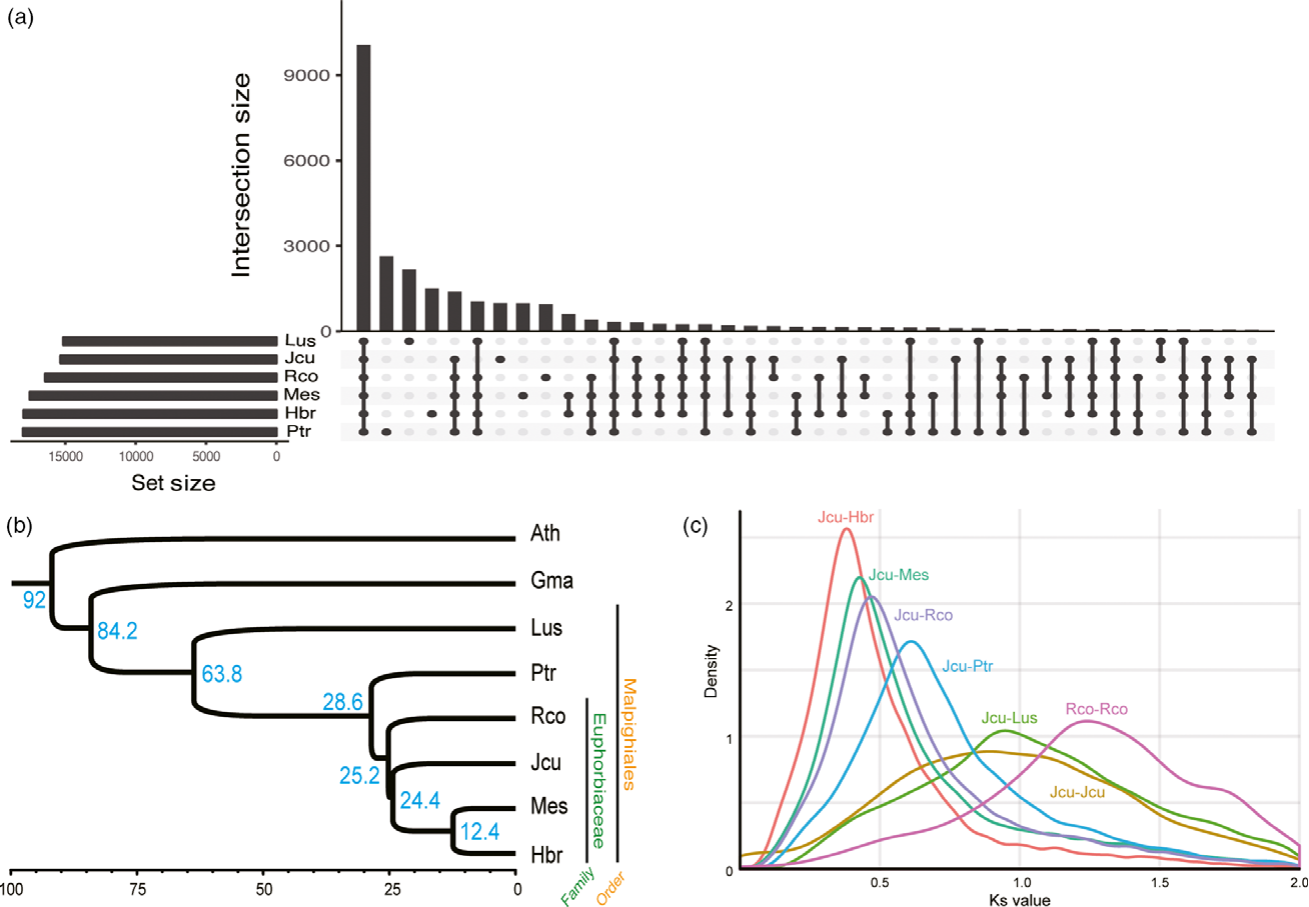
Phylogenetic analysis in Malpighiales order. (a) Upset plot of orthologous gene groups among six species in the Malpighiales order. Orthologous gene groups were clustered by OrthoMCL v2.0.9. (b) Phylogeny tree using 67 single‐copy orthologues from eight species. Four species in Euphorbiaceae family (*J. curcas*,* M. esculenta*,* H. brasilensis* and *R. communis*), two species in Malpighiales order (*P. trichocarpa* and *L. usitatissimum*) and two outgroups (*G. max* and *A. thaliana*) were included for the analysis. The tree was constructed by baysian method using BEAST with JTT+G as the best‐fit model. The root divergence time was set to the estimated divergence time between Brassicales and Fabales (~92 mya). The numbers in blue indicate estimated divergent time of each node (million years ago). (c) Ks value distribution of the species in the Malpighiales order. Ks value was calculated between Jcu and Mes, Rco, Ptr and Lus and within Jcu and Rco. (Ath: *Arabidopsis thaliana*, Gma: *Glycine max*, Hbr: *Hevea brasilensis*, Lus: *Linum usitatissimum*, Mes: *Manihot esculenta*, Jcu: *J. curcas*, Rco: *Ricinus communis* and Ptr: *Populus trichocarpa*.)

Orthologous gene groups shared among six species in the order Malpighiales, including black cottonwood (*Populus trichocarpa*), cassava (*Manihot esculenta*), castor bean (*Ricinus communis*), flax (*Linum usitatissimum*), physic nut (*Jatropha curcas*) and rubber tree (*Hevea brasilensis*) were clustered using the gene models (Figure 1a). We constructed a phylogenetic tree using 67 conserved, single‐copy orthologs from the six species with *Glycine max* and *Arabidopsis thaliana* serving as the outgroup (Figure 1b). The results of phylogenetic analysis are consistent with the genome‐wide Ks value distributions among major species in the Euphorbiaceae family (Figure 1c) and the general phylogeny of eudicots provided by PLAZA3.0 (Proost *et al*., 2015). Using the divergence time between Brassicales and Fabales of ~92 million years ago (mya) as a calibration point, we estimated that divergence of the Euphorbiaceae family occurred ~63.8 mya (Figure 1b), which is similar to the previous estimates of 65.6 mya (Wu *et al*., 2015) and 57.7 mya (Tang *et al*., 2016). This value is also consistent with the divergence time (~62.8 mya) estimated from another phylogenetic tree constructed by baysian method using 42 orthologous genes based on synteny, which estimated the divergence times of flax (67.9 mya), poplar (62.8 mya), castor bean (54.2 mya), Jatropha (54.0 mya), and cassava and rubber tree (35.7 mya) (Figure S4). The tree we constructed provides overall divergence time of the economically important crops in the order Malpighiales while previous genome papers of major Malphighiales crops missed at least one species out in phylogenetic analyses (Tang *et al*., 2016; Wang *et al*., 2014b; Wu *et al*., 2015).

**Figure 2:**
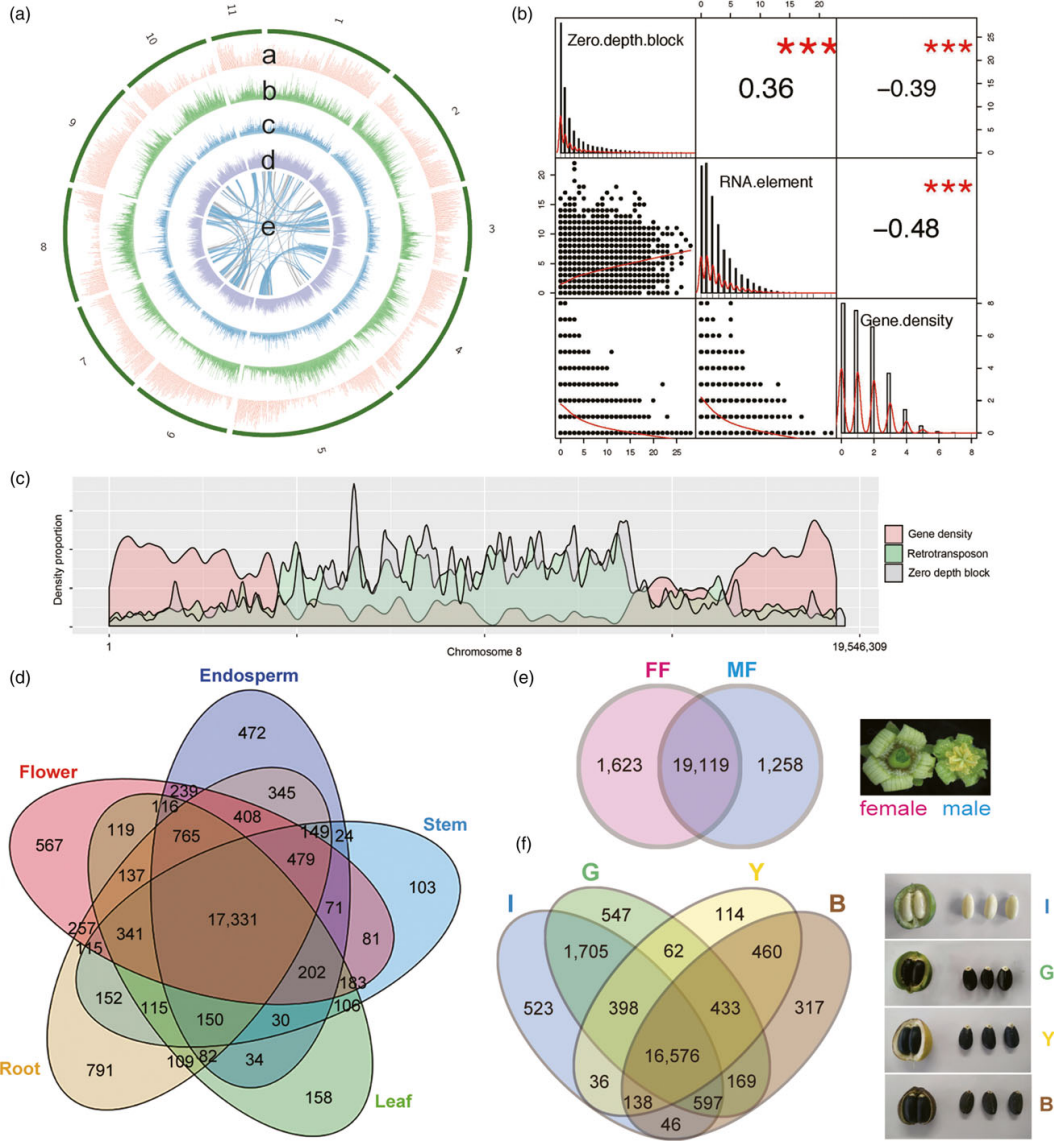
Genome structure of *J. curcas* CN. (A) Gene density (a, red) are depicted in the outer circle of the circular map. The middle circle shows the distribution of retrotransposon (b, green), DNA transposon (c, blue) and other repeats (d, purple). The inner circle represents self synteny blocks in Jatropha by grey lines and the synteny regions with two or more paralogous blocks are highlighted by blue lines (e). A 10 kbp window was applied to repeats (b‐d). (B) Correlation matrix presenting the correlation among Illumina zero mapping depth block, retrotransposon and gene density displayed along the diagonal. Pearson correlation coefficients between the traits are shown on the right of the diagonal. The correlation significance level is ***P < 0.001. The statistical analysis was performed by R package, PerformanceAnalytics. (C) Distribution of genes, retrotransposon and Illumina zero depth block on chromosome 8. (D) Venn diagram of shared gene clusters among five different tissues, endosperm, stem, leaf, root and flower in *J. curcas* CN. (E) Venn diagram of shared gene clusters between female and male flowers. MF indicates male flower and FF indicates female flower. (F) Venn diagram of shared gene clusters among four different seed development stages. I, G, Y and B indicate seed endosperms from immature fruit, green fruit, yellow fruit and brown fruit, respectively.

Although Jatropha diverged later than castor bean, Jatropha has a broader peak in Ks distribution than castor bean (Figure 1c), suggesting that Jatropha likely experienced more dynamic evolution than castor bean after speciation. Syntenic blocks with two or more paralogous synteny pairs (~72% of all synteny pairs) were identified multiple times in Jatropha, as was found in *R. communis* (Figure 2a; Chan *et al*., 2010). These results support the notion that all dicots (including members of the Euphorbiaceae family) underwent a paleo‐hexaploidization rather than a single duplication event (Jaillon *et al*., 2007; Velasco *et al*., 2007).

### Transcriptome analysis

**Figure 3:**
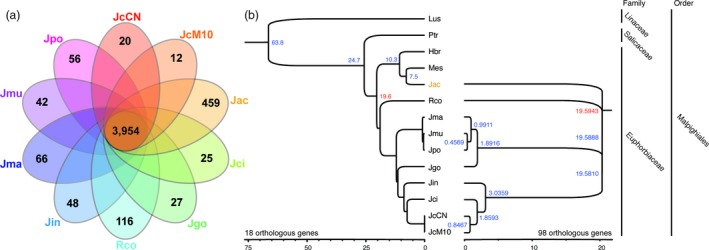
Divergence of nine Jatropha species. (a) Venn diagram of shared orthologous gene group of nine *Jatropha* species and *Ricinus communis*. (b) Phylogenic tree of nine Jatropha species with flex, poplar, rubber tree and cassava in the Malpighiales order. A phylogenetic tree of nine *Jatropha* species with castor bean as outgroup was contructed based on 98 true orthologous genes using BEAST v.1.8.4 by bayesian method (the right tree). The estimated divergence time between Ptr and Lus (Fig. 1b) was used as a calibration point. To clarify phylogenetic location of *J. aconitiforia,* a phylogenetic tree of *Jatropha* species with four relative species in Malpighiales order was constructed (the left tree). Out of 98 genes, 15 gene orthologs were selected to construct the tree using phyml v.3.1 by maximum likelihood method. The divergence time was estimated by MCMCTree based on the estimated divergence time between Hbr and Rco. Hbr: *Hevea brasilensis*, Lus: *Linum usitatissimum*, Mes: *Manihot esculenta*, JcCN: *J. curcas* var. CN, JcM10: *J. curcas* var. M10, Jac: *J. aconitiforia*, Jci: *J. cineria*, Jgo: *J. gossypifolia*, Jin: *J. intergerrima*, Jma: *J. macrantha*, Jmu: *J. multifida* and Jpo: *J. podagrica*, Rco: *Ricinus communis*, Ptr: *Populus trichocarpa*.

The transcriptome analysis was performed using leaf tissues of nine Jatropha species and castor bean (*Ricinus communis*) to identify orthologous gene groups (Data S1, Figure S5, Table S5). The number of orthologous gene groups shared by all 10 species was 3954 (Figure 3a). *J. aconitifolia* had the greatest number of specific orthologous gene groups (459), with even more than castor bean (116). We constructed a phylogenetic tree using 98 highly conserved gene orthologs from nine *Jatropha* species and castor bean, finding that *J. aconitifolia* did not group with the other *Jatropha* species (Figure 3b). To further clarify the taxonomy of *J. aconitifolia*, we constructed a phylogenetic tree in the order Malpighiales, including flax, poplar, rubber tree and cassava. *J. aconitifolia* was grouped with cassava (*Manihot esculenta*), which is also in the Euphorbiaceae family, with a divergence time from *Jatropha* species estimated to be ~19.6 mya (Figure 3b). The phylogenetic tree shows *J. cineria* is the closest species to *J. curcas* among the *Jatropha* species examined.

RNA raw reads from different tissues in *J. curcas* CN were mapped to the gene model for tissue specific expression (Figure 2d,e,f, Data S3) (Feng *et al*., 2012; Grabherr *et al*., 2011). An overall comparison among five tissue types (endosperm, stem, leaf, root and flower tissue) revealed that a total of 17 331 genes were shared by all five tissue types (Figure 2d). More genes were specifically expressed in female (1623) versus male flowers (1258) (Figure 2e). Among the 1538 differentially expressed genes (DEGs) detected between female and male flowers, transcription factor (TF) activity was one of the most highly enriched GO terms (Figure S6, Data 3) (Maere *et al*., 2005). Based on homology to sequences in the database plantTFDB (Guo *et al*., 2008), 99 DEGs were identified as TF genes (Table S8). AP2‐EREBP was the most highly enriched differentially expressed TF (DETF), followed by MADS and WRKY. Female flowers had many more up‐regulated DETFs (76 DETFs) than male flowers (23 DETFs). Most AP2‐EREBP DETFs were up‐regulated in female flowers, which is reminiscent of the enrichment of AP2‐EREBP in embryo sac‐enriched tissue samples in maize (Chettoor *et al*., 2014). The most highly enriched DETF in male flowers was MADS, with 7 of 15 MADS DETF. This is consistent with the finding that, in Arabidopsis and maize, MADS transcripts are over‐represented in male pollen, which supports an ancient role for these genes in the male gametophyte (Chettoor *et al*., 2014; Kwantes *et al*., 2012; Verelst *et al*., 2007). The second most enriched DETF in male flowers, WRKY, has been reported to be required for male gametogenesis in Arabidopsis (Guan *et al*., 2014). The ratio of female‐to‐male flowers greatly affects seed yield in Jatropha, a monoecious tree, making flowering in *J. curcas* a source of great interest (Wijaya *et al*., 2009). Chen *et al*. uncovered transcriptional changes in inflorescence buds of *J. curcas* under cytokinin treatment that can increase the number of female flowers, and Xu *et al*. profiled DEGs over six different developmental phases in the floral primordia of this species (Chen *et al*., 2014; Xu *et al*., 2016). Our data for DETFs between female and male flowers, along with the previous transcriptome profiling of floral buds and primordia, should help elucidate the sex differentiation mechanism in *J. curcas*.

### Lipid biosynthesis in Jatropha

**Figure 4:**
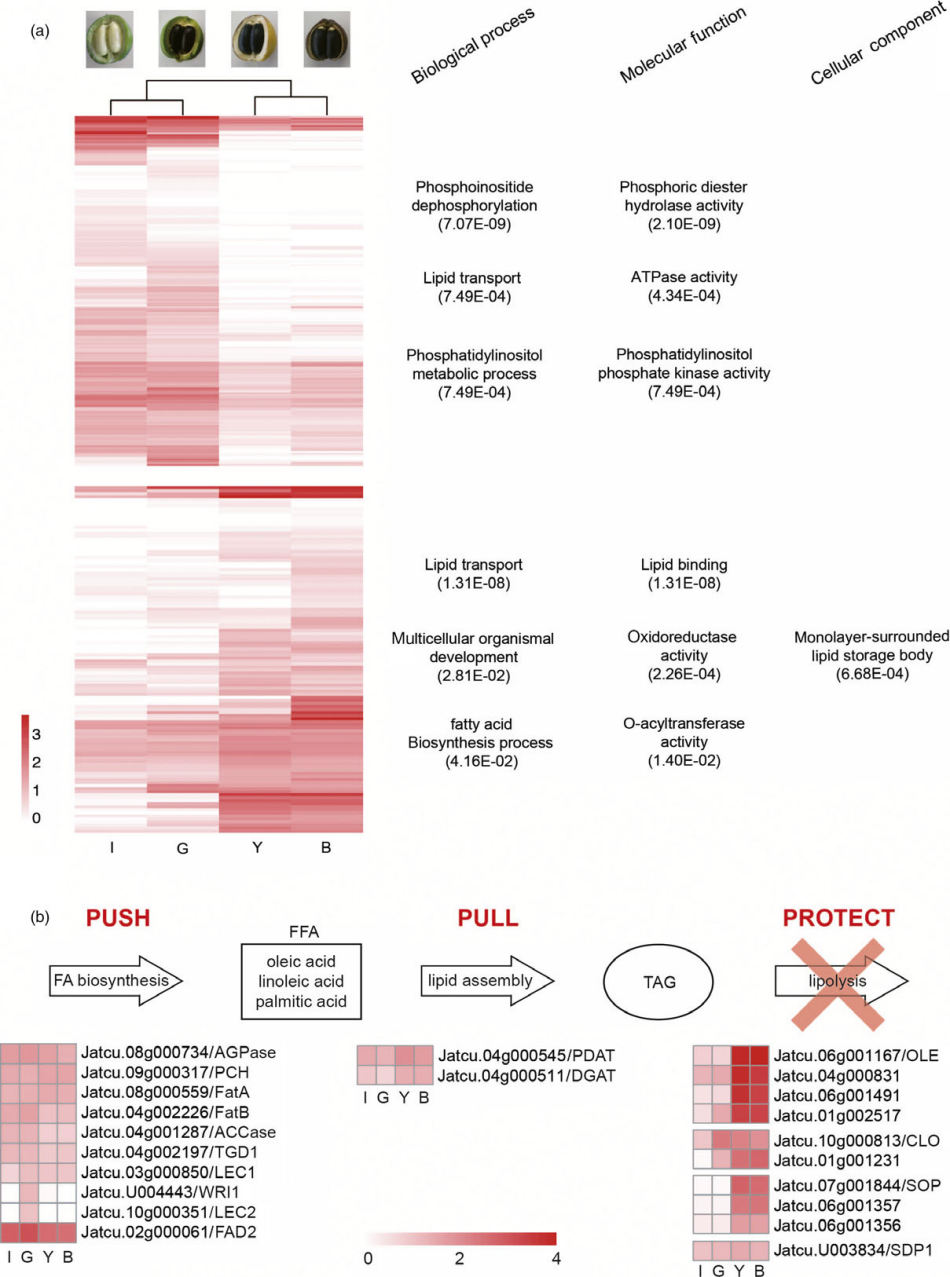
Heatmaps of acyl lipid genes in four different Jatropha endosperms. (a) DEGs are clustered into two groups based on expression patterns (higher expression in early stage or late stage). The most significantly enriched GO terms of two groups are indicated on the right side. The color scale on the bottom left demonstrates log_10_RPKM values. I: immature fruit, G: green fruit, Y: yellow fruit, B: brown fruit. (b) The expressions of homologs involved in TAG accumulation are listed under ‘Push’ the biosynthesis of fatty acid, ‘Pull’ TAG assembly and ‘Protect’ the prevention of lipolysis. ACCase: Acetyl‐CoA carboxylase, AGPase: ADP‐glucose‐pyrophosphorylase, CLO: Caleosin, DGAT: Acyl‐CoA:diacylglycerol acyltransferase, FatA: Acyl‐ACP thioesterase A, FatB: Acyl‐ACP thioesterase B, LEC: LEAFY COTYLEDON 1, WRI1: WRINKLED1, WRI2: WRINKLED2, FAD2: Oleoyl‐ACP desaturase2, OLE: Oleosin, PCH: Palmitoyl‐CoA hydrolase, PDAT: Phospholipid:diacylglycerol acyltransferase, SDP1: Sugar‐dependent 1, SOP: Steroleosin, STO: Steroleosin, TAG: Triacylglycerol, TGD1: Trigalactosyldiacylglycerol1. The color scale on the bottom demonstrates log_10_RPKM values.

`We found that 16 576 genes were commonly expressed in endosperms from immature, green, yellow and brown fruits (IF, GF, YF and BF) (Figure 2f, Data S3). Endosperms from the early stage (IF and GF) had more stage‐specific gene expressions than the late stage (YF and BF). Based on homology to 775 genes in 24 acyl lipid sub‐pathways in Arabidopsis (http://aralip.plantbiology.msu.edu/pathways/pathways) (Li‐Beisson *et al*., 2010), 862 putative acyl lipid biosynthesis genes in *J. curcas* CN were identified, of which 305 genes were differentially expressed among the four endosperm tissues (Table S9). Of the 24 sub‐pathways, Fatty Acid (FA) Elongation & Was Biosynthesis was the most highly enriched sub‐pathway, followed by Phospholipid Signaling and Triacylglycerol Biosynthesis (Table S10). Most sub‐pathways enriched in Jatropha were also enriched in castor bean, oil palm, soybean and sesame (Chan *et al*., 2010; Li *et al*., 2014; Singh *et al*., 2013; Wang *et al*., 2014a). Based on the expression patterns of the putative acyl lipid genes, 305 DEGs were clustered into two groups; DEGs up‐regulated in early stage (IF and GF) and DEGs up‐regulated in late stage (YF and BF) (Figure 4a, Table S11). In early stage, GO terms related to lipid biosynthesis, such as phosphoinositide dephosphorylation (GO:0046839), phosphatidylinositol metabolic process (GO:0046488), phosphoric diester hydrolase activity (GO:0008081) and phosphatidylinositol phosphate kinase activity (GO:0016307), were enriched, indicating that Jatropha uses phospholipids as acyl donors for TAG synthesis (Dahlqvist *et al*., 2000). In late stage, GO terms related to lipid storage, such as lipid transport (GO:0006869), lipid binding (GO:0008289), and monolayer‐surrounded lipid storage body (GO:0012511), were enriched.

Oil seed content and quality are determined by multiple metabolic levels including fatty acid synthesis (‘Push’), TAG assembly (‘Pull’) and lipolysis (‘Protect’) (Figure 4b) (Napier *et al*., 2014). The production of oleic, linoleic and palmitic acid, the main constituents of Jatropha seed oil, is catalysed by the enzyms Acyl‐ACP thioesterase A and B (FatA and FatB) and Palmitoyl‐CoA hydrolase (PCH) (Jones *et al*., 1995; Voelker, 1996). Homologs of FatA and B (*Jatcu.08 g000559* and *Jatcu.04 g002226*) and PCH (*Jatcu.09 g000317*) were detected as DEGs during four stages of fruit development. TAG, the major component of seed storage oil, is synthesized by two enzymes, diacylglycerol acyltransferase (DGAT) and phospholipid:diacylglycerol acyltransferase (PDAT) in Arabidopsis (Li‐Beisson *et al*., 2010; Zhang *et al*., 2009, 1). The PDAT homolog (*Jatcu.04 g000545*) had much higher expression level at all stages than the DGAT homolog (*Jatcu.04 g000511*), suggesting TAG synthesis is mainly catalysed by PDAT in Jatropha. In castor bean, an oil seed crop in the Euphorbiaceae family, the expression of DGAT was much higher than that of PDAT (Brown *et al*., 2012), while PDAT mainly catalyses TAG synthesis in sesame, which has much higher oil content than soybean, rapeseed and peanut (~55% of dry seed), (Wang *et al*., 2014a; Wei *et al*., 2013). Wang *et al*. (2013) showed PDAT had significantly higher expression than DGAT in sesame and the determination of different oil content begins in the early stage of seed development. This agrees well with our data that GO terms related to phospholipid were enriched and more stage‐specific gene expressions were detected in the early stages of Jatropha fruit development (Figures 2f and 4a). Homologs of oleosin, caleosin and steroleosin, encoding oil body proteins that prevent the breakdown of TAG in the cytosol in oil seed plants, showed consistently high expression during all four stages. Particularly, homologs of oleosin (*Jatcu.06 g001067*,* Jatcu.04 g000831*,* Jatcu.06 g001491* and *Jatcu.01 g002517*) had much higher expression levels at late stages of seed development than homologs of caleosin and steroleosin, indicating the prevention of lipolysis by oleosin allows Jatropha to accumulate high levels of oil in kernel (~63%) along with high level of oil biosynthesis (Akbar *et al*., 2009; Tzen *et al*., 1992). Here, we provide target genes for genetic engineering to improve seed oil contents and quality of Jatropha over the ‘Push’, ‘Pull’ and ‘Protect’ integrated concept of TAG accumulation.

### Phorbol ester biosynthesis in Jatropha

**Figure 5:**
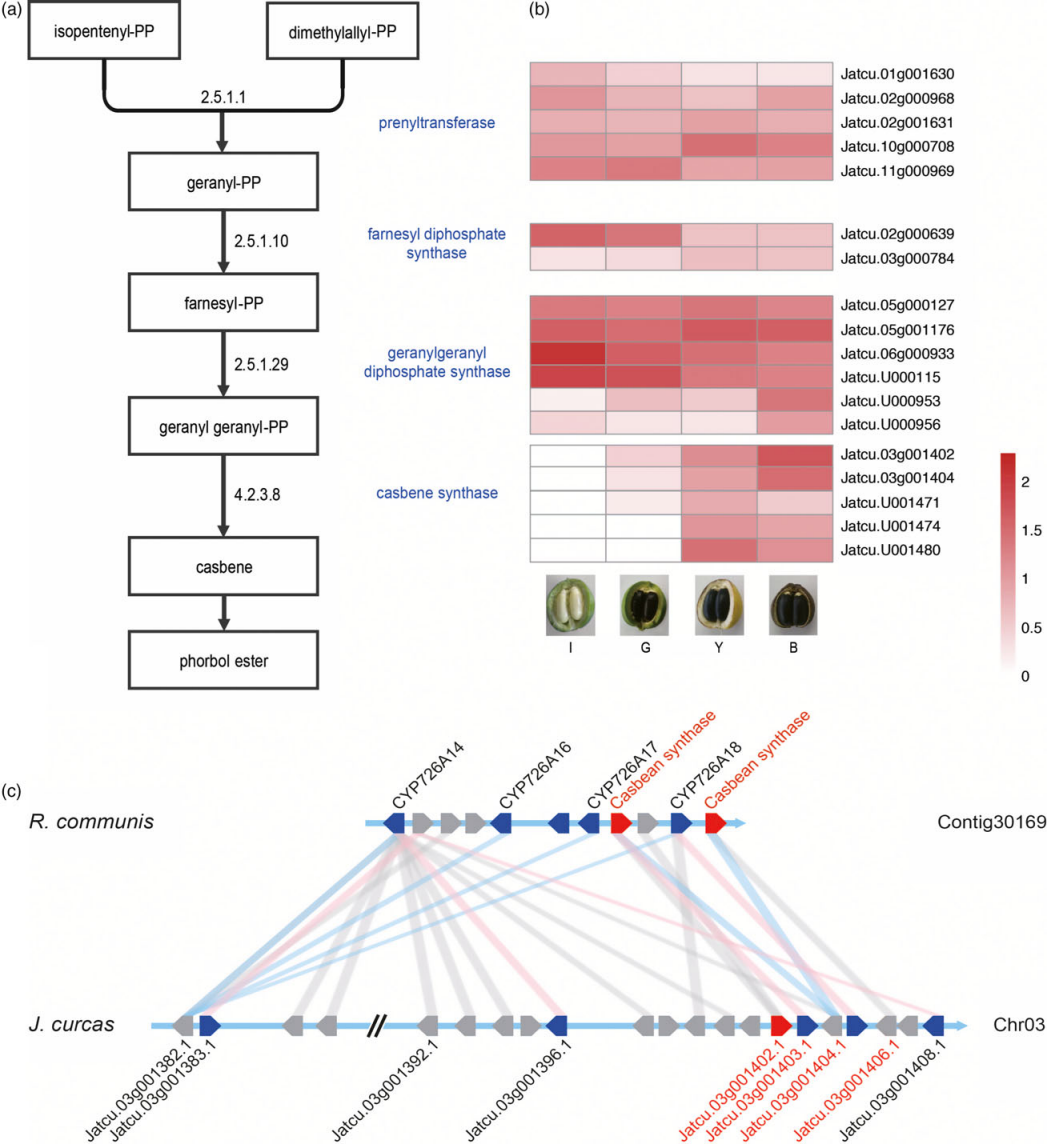
Phorbol ester biosynthesis in *J. curcas*. (a) Phorbol ester biosynthesis pathway. The names of key enzymes are indicated in blue and EC numbers are indicated on the arrows. (b) Heatmaps of candidate key enzymes in PE biosynthesis pathway. The color scale on the bottom right demonstrates log_10_RPKM values. I: immature fruit, G: green fruit, Y: yellow fruit, B: brown fruit. (c) Synteny block of a physical cluster of diterpenoid biosynthetic genes between *J. curcas* (Jcu) and *R. comunis* (Rco). Syntenic relationship is indicated by grey lines between the genes indicated by pentagons. Functionally characterized casbene synthases and cytochrome P450s in Rco are indicated by red and blue pentagons, respectively, on contig30169. The Jcu genes with the highest homology to the Rco genes are linked by blue lines. The Jcu genes with Jcu‐Rco Ks value within 0.32 and 0.63 (Table S15) are indicated by blue pentagons on Chromosome 3. Functionally characterized casbene synthase in Jcu (*Jcu03g001402.1* or *JcCASA163*) is indicated by a red pentagon. The Rco genes with the highest homology to the Jcu genes are linked by pink lines. Average mapping depths of the physical cluster are 5.08× of Pacbio and 66.87× of Illumina paired end.

Phorbol ester (PE), a major toxic compound in *Jatropha* seed cake, is a diterpenoid found in some members of the Euphorbiaceae family (Figure 5a). Based on homology to genes involved in PE biosynthesis in the Euphorbiaceae family, 26 genes were found to be related to PE biosynthesis in *J. curcas* CN, of which 18 genes were identified as DEGs among four different stages of seed development (Figure 5b, Data S3) (Costa *et al*., 2010). Casbene is a precursor to PEs, and the down‐regulation of casbene synthase can dramatically reduce PE levels in Jatropha seeds (Li *et al*., 2015). We identified 10 casbene synthase gene homologs in the Jatropha assembly, including five genes with little expression in the endosperm and five that were much more highly expressed at the later stages of fruit development than at the earlier stages (Table S12). *Jatcu.03 g001402* had the highest expression level at the last stage of fruit maturity (BF); RNAi of this gene reduced PE contents to 28% of control levels in *J. curcas* (Li *et al*., 2015). A physical cluster of diterpenoid biosynthesis genes, including casbene synthase genes, was identified on Jatropha chromosome 3, as found in *R. communis* (Figure 5c). The gene cluster in Jatropha has four casbene synthase homologs but two in *R. communis* (Figure 5c). *Jatcu.03 g001402* and *Jatcu.03 g001404* had the highest expression levels among casbene synthase homologs in the cluster. Li *et al*. (2015) showed that down‐regulating *Jatcu.03 g001402* (*JcCASA163*) and *Jatcu.U001474* (*JcCASA168*) expression reduced PE levels to 15% of the wild type. Here, we identified other candidate casbene synthase genes (*Jatcu.03 g001404* and *Jatcu.U001480*), which had higher transcript levels than *Jatcu.U001474*; down‐regulating the expression of these genes in conjunction with *Jatcu.03 g001402* might yield Jatropha seeds with little or no PE (Table S12).

## Discussion

We constructed a 339.4 Mbp assembly of *J. curcas* CN (82% of the estimated genome size) with a superscaffold N50 length of 15.4 Mbp (Carvalho *et al*., 2008). The N50 lengths of contigs (1.0 Mbp) containing no ambiguous sequences (Ns), scaffolds (1.5 Mbp) and superscaffolds (15.4 Mbp) were much improved compared with the previously reported Jatropha genome assemblies by Wu *et al*. (2015) and Hirakawa *et al*. (2012) (JAT_r4.5, http://www.kazusa.or.jp/jatropha/) (Table S13) (23, 52, 53). We assembled the Jatropha genome using only Illumina short reads consisting of 48.5 Gbp of paired‐end reads and 133.5 Gbp of mate pair reads, resulting in 3710 scaffolds totalling 319 Mbp, which is highly fragmented compared with the assembly using PacBio long reads and Illumina short reads together (917 scaffolds). To investigate the differences between the two assemblies, we mapped Illumina paired‐end reads against the PacBio assembly. Although 95.61% of paired‐end reads were properly mapped, the PacBio assembly had 48 162 blocks spanning 3 154 711 bp with zero mapping depth of Illumina paired‐end reads (Figure 2c and Table S14). Although the blocks cover only ~1% of the assembly, they are distributed throughout the genome, which explains the fragmentation of the assemblies obtained from Illumina short reads. We assembled a higher quality Jatropha genome using PacBio long reads and Illumina short reads together compared with any other Jatropha assemblies obtained using the Sanger method, Roche/454, Illumina GA, or HiSeq (Table S13; Hirakawa *et al*., 2012; Sato *et al*., 2011; Wu *et al*., 2015). The distribution of the blocks with zero mapping depth of Illumina paired‐end reads was positively correlated with RNA transposons, and gene density was negatively correlated with RNA transposons (Figure 2b). A negative correlation between the density of class I retrotransposons and gene density has also been observed in sorghum (Paterson *et al*., 2009) and maize (Schnable *et al*., 2009), but not in Arabidopsis (Wright *et al*., 2003) or rice (Tian *et al*., 2009), where DNA transposons and gene density are negatively correlated. RNA transposon‐rich regions might not be sequenced due to biases during library construction, which is a limitation of Illumina sequencing. The presence of repeat sequence related features in a genome, such as inverted repeats, microsatellite DNA, high‐ and low‐GC regions, and secondary structures in single‐stranded DNA, can result in bias in Illumina sequencing, but in Jatropha, the presence of RNA transposable elements likely causes the bias in Illumina sequencing (Harismendy *et al*., 2009; Nakamura *et al*., 2011; Ross *et al*., 2013; Star *et al*., 2016; Stein *et al*., 2010).

Jatropha seeds contain up to 40% oil consisting of ~75% unsaturated fatty acids with a high level of linoleic acid (~47%) which is favourable oil composition for biodiesel production (Adebowale and Adedire, 2006; Gübitz *et al*., 1999). Seed storage lipid was increased up to 30% compared with control by silencing *SDP1* in Jatropha using RNAi technology, due to blockage in TAG degradation (Kim *et al*., 2014). The quality of seed oil was greatly improved in RNAi transgenic plants of *FAD2*, a major enzyme responsible for converting oleic acid to linoleic acid (Qu *et al*., 2012). The proportion of oleic acid in Jatropha seed oil was enhanced to >78% compared to the control plant (~37%), which agree with consistently high expression of *FAD2* over four seed developmental stages in our transcriptome data (Figure 4b). Through virus‐induced gene silencing (VIGS) system, co‐silencing of *KASII* and *FatB* changed fatty acid composition in Jatropha seed oil (Ye *et al*., 2009). The quantity and quality of seed oil for biodiesel production can be much improved by genetic engineering on multiple metabolic levels instead of single‐gene strategies (Napier *et al*., 2014). In this study, through intensive transcriptomic analysis based on the refined genome assembly, the target homologous genes for genetic engineering and their expression profiles were identified in the biosynthesis of fatty acid (‘Push’) and TAG (‘Pull’), and the prevention of lipolysis (‘Protect’) in Jatropha (Figure 4b). Genomic information pertaining to the DEGs in lipid biosynthesis among four different stages of seed development would provide a basis for optimization of the ‘Push’, ‘Pull’ and ‘Protect’ integrated concept of TAG accumulation in Jatropha seed, as well as improvement of oil quality for biodiesel production.

The efficiency of Jatropha seed as a source for biodiesel has been underestimated compared to other oil seed crops (Gerbens‐Leenes *et al*., 2009). The efficiency of Jatropha seed oil based on its actual yield produced by smallholders under rain‐fed conditions compared with the those of soybean and rapeseed cultivated under additional irrigation deserves correction (Jongschaap *et al*., 2009); indeed, it shows better yield under better irrigation conditions in semiarid areas (de Carvalho *et al*., 2015). Furthermore, the toxicity of seed cake, which has similar energy potential to seed oil, makes the efficiency of Jatropha oil undervalued (Jongschaap *et al*., 2009). Except for some accessions from Central America, all parts of the Jatropha plant are toxic, including *J. curcas* CN, one of the most productive Jatropha varieties. A quantitative trait locus for PE contents was identified in the genomic region containing *Jatcu.03 g001402* (*JcCASA163*) encoding casbene synthase, the most responsive enzyme for PE contents in Jatropha seeds (Figure 5c; King *et al*., 2013; Li *et al*., 2015). However, further analysis of additional candidate genes for casbene synthase and syntenic regions between Jatropha and castor bean has been limited due to the lack of high‐quality genomic data combined with intensive transcriptome analysis. In the physical cluster of diterpenoid biosynthesis genes we identified in this study, based on the Ks distribution between *J. curcas* and *R. communis*, five Jatropha genes (Ks value between 0.32 and 0.63) originated from a common ancestor (Figure S7 and Table S15) and, after the divergence, the other genes in the cluster have diverged more dynamically in Jatropha than in castor bean. Although PE has been reported to diffuse into the endosperm from the tegmen, the expression levels of candidate casbene synthase genes in endosperm significantly differ among developmental stages (Figure 5b) (King *et al*., 2013). Here, we reported two casbene synthases in the cluster and three in other regions of the genome to be detected as DEGs among four seed developmental stages (Li *et al*., 2015). Identification of target genes for genetic engineering would facilitate development of elite cultivars with little or no PE, increasing the efficiency of Jatropha oil.

We reported the transcriptome data from leaf tissues of nine *Jatropha* species (S5). Existence of natural hybrid complexes has been reported in the genus *Jatropha* (Dehgan and Webster, 1978; Prabakaran and Sujatha, 1999). Interspecies crossing has been recommended for genetic studies and breeding programs due to low DNA variation in *J. curcas* (Divakara *et al*., 2010; Yue *et al*., 2013). Although relative species, such as *J. intergerrima* and *J. gossypifolia*, have been used for hybrid breeding and genetic studies (Divakara *et al*., 2010; Liu *et al*., 2011; Sujatha and Prabakaran, 2003; Sun *et al*., 2012; Wang *et al*., 2011), phylogenetic analysis using the transcriptome data suggests that *J. cineria,* a genetically closer species to *J. curcas* (diverged 0.85 mya ago), is a good candidate for interspecific hybridization with *J. curcas* (Figure 3b), avoiding linkage disequilibrium likely caused by genetic distance in the previous interspecific crosses (Liu *et al*., 2011; Sun *et al*., 2012; Wang *et al*., 2011). The transcriptome data will serve as valuable genetic resources to improve *Jatropha* cultivars through increasing genetic diversity and importing favoured alleles. Phylogenetic analysis clarified the taxonomic confusion of *J. aconitifolia* caused by old and incorrect naming. The correct name of this species is *Cnidoscolus aconitifolius* and, based on botanical studies, the genus *Cnidoscolus* belongs to the tribe Manihoteae of the Euphorbiaceae family with the genus Manihot, which agrees very well with the phylogenetic tree (Figure 3b; Miller and Webster, 1962; Ross‐Ibarra and Molina‐Cruz, 2002; Tokuoka, 2007). The robust phylogenetic tree we constructed clarified the taxonomy in the order Malpighiales enabling estimation of divergence times among nine *Jatropha* species and economically important crops in the Euphorbiaceae family.

Jatropha is primarily grown in developing countries. This plant can be vegetatively propagated, and plants currently grown in Africa, Asia and South America are nearly clonal, causing a narrow genetic variation, except in Mesoamerica, the origin of this species (Montes Osorio *et al*., 2014; Pecina‐Quintero *et al*., 2014; Sun *et al*., 2008). Attributed to the lack of elite cultivars lacking toxic compounds, Jatropha has not performed to the yield potential expected by smallholders. As a non‐edible and monoecious biodiesel crop, physic nut has much potential to be improved by genetic engineering as well as conventional breeding. The high‐quality reference genome sequence data obtained in the current study should boost molecular breeding efforts for Jatropha improvement, which should help double the energy yield by increasing seed oil content and enabling seed cake to be used as animal feed.

## Materials and Methods

### Genome assembly

The *J. curcas* CN genome was sequenced using two platforms, PacBio RS II and Illumina HiSeq2000, with five libraries of 200 bp for paired‐end reads (SRR5974850), 5 kbp (SRR5974847) and two 10 kbp for mate pairs (SRR5974845 and SRR5974848), and 20 kbp for PacBio (SRR5974849) (Table S1). PacBio long reads were assembled into contigs using Falcon v0.3.0 after error correction with Canu v1.0 (Figure S1; Chin *et al*., 2016; Koren *et al*., 2017). The contigs were scaffolded using SSPACE v3.0 and anchored into pseudochromosomes with ALLMAPS using the two genetic maps (Boetzer *et al*., 2011; Tang *et al*., 2015). The gaps in the superscaffolds were filled using Illumina paired‐end reads with Gapfiller v1.10 (Boetzer and Pirovano, 2012). Illumina paired‐end reads were filtered using NGS QC Toolkit and mapped against superscaffolds to calculate mapping depth and heterozygosity using BWA (Tables S4 and S14) (Li and Durbin, 2009; Patel and Jain, 2012). The numbers of Illumina zero mapping depth blocks, retrotransposons and genes in every 10 kbp were counted throughout the genome and Pearson correlation coefficient between the traits were calculated using R package, PerformanceAnalytics (Figure 2b). Length distribution and the frequency of 5‐mers in Illumina zero depth blocks were counted using an in‐house Python script (Figure S8 and Table S16). Genome assembly and annotation data are available at http://plantgenomics.snu.ac.kr/.

### Genome annotation

*De novo* and homology‐based gene prediction were performed via the MAKER annotation pipeline based on transcriptome data from five different tissues (leaf, root, flower, stem and endosperm) of *J. curcas* CN (Data S1; Cantarel *et al*., 2008). Before gene prediction, RepeatMasker v. open‐4.0.5 was used to annotate repeat sequences on the genome assemblies using a library constructed using RepeatModeler, LTRharvest and LTRdigest (Ellinghaus *et al*., 2008; Smit *et al*., 2014; Steinbiss *et al*., 2009; Tarailo‐Graovac and Chen, 2009). An initial gene model constructed with the MAKER pipeline was used to train AUGUSTUS model parameters (Stanke *et al*., 2006). Using the initial gene model, the gene prediction pipeline was re‐run against the repeat masked and unmasked genome assemblies (Table S7). A set of the resulting high‐confidence genes was annotated using Interproscan5 (Quevillon *et al*., 2005). GO classification of Jatropha genes was visualized using WEGO (Figure S9; Ye *et al*., 2006). The CEGMA and BUSCO programs were used to evaluate the completeness of the gene space in the assembly (Table S3; Parra *et al*., 2007; Simão *et al*., 2015). For CEGMA, 248 core eukaryotic genes were mapped, and 1440 embryophyta genes were used for BUSCO. Transcriptome data from five *J. curcas* tissues were mapped to the assembly using BLAT, and transcripts with 90% or higher identity (aligned length/total length) were counted as properly mapped transcripts (Table S5; Kent, 2002). Simple sequence repeats were predicted based on the assembled scaffolds using GMATo v1.2 with default parameters (Tables S17 and S18) (Wang *et al*., 2013). Synteny blocks were detected among eight species, including *A. thaliana*,* G. max*,* H. brasiliensis*,* J. curcas*,* L. usitatissimum*,* M. esculenta*,* P. trichocarpa* and *R. communis* (www.phytozyme.net), using MCScanX, and Ks values of the homologs within collinearity blocks among Malpighiales species were calculated using a Perl script, add_ka_and_ks_to_collinearity.pl, in the MCScanX package (Figures 1c and S7) (Wang *et al*., 2012).

### Phylogenetic analysis

Orthologous gene groups shared among *A. thaliana*,* G. max*,* H. brasiliensis*,* J. curcas*,* L. usitatissimum*,* M. esculenta*,* P. trichocarpa* and *R. communis* were clustered by OrthoMCL using the gene models, and a Upset plot was constructed using six species in Malpighiales (Figure 1a; Lex *et al*., 2014; Li *et al*., 2003). A phylogenetic tree was constructed using 67 conserved, single‐copy gene orthologs among eight species (*A. thaliana*,* G. max*,* H. brasiliensis*,* J. curcas*,* L. usitatissimum*,* M. esculenta*,* P. trichocarpa* and *R. communis*) using BEAST 1.8.4 (Figure 1b; Heled and Drummond, 2010). The protein sequences were aligned using Muscle v3.8.31 (Edgar, 2004). JTT+G was selected as the best‐fit model by Prottest (Abascal *et al*., 2005). The divergence time (92 mya) between Brassicales (including *A. thaliana*) and Fabales (including *G. max*) was used as a root time calibration point (Gandolfo *et al*., 1998). A phylogenetic tree was constructed using 42 orthologous gene sequences based on synteny identified using MCScanX as described above (Figure S4). To construct a phylogenetic tree of the nine *Jatropha* species, the non‐redundant CDS of *J. curcas* CN clustered by CD‐HIT v4.6.4 (Li and Godzik, 2006) was mapped by blastp (Camacho *et al*., 2009) against those of nine *Jatropha* species and castor bean with an e‐value of 1e^−10^ (Figure 3b). Ninety‐eight true orthologous genes were selected when the best hits from each species were included in the same orthologous gene groups (clustered by OrthoMCL v2.0.9) (Li *et al*., 2003) and the orthologous genes had no length polymorphism. Among the 98 gene orthologs, 18 genes were shared by four other Malpighiales species (*H. brasiliensis*,* L. usitatissimum*,* M. esculenta* and *P. trichocarpa*). The protein sequences were aligned using Muscle v3.8.31, and the tree was constructed using PhyML v3.1. The divergence time was estimated using the MCMCTree program from PAML package 4.9e based on a calibration point between *L. usitatissimum* and *P. trichocarpa* of ~19.5943 mya (Figures 1b and 3b; Yang, 2007).

### Lipid and phorbol ester biosynthesis in the Euphorbiaceae family

Putative acyl lipid genes in *J. curcas* CN were identified by blastp with e‐value 1e^‐10^ against genes in the 24 acyl lipid sub‐pathways in Arabidopsis (http://aralip.plantbiology.msu.edu/pathways/pathways) (Tables S10 and S11; Li‐Beisson *et al*., 2010). DEGs were clustered into two groups depending on whether they were expressed higher in early (IF and GF) or late stages (YF and BF), and the heatmap was constructed based on log_10_RPKM values for lipid biosynthesis genes using the pheatmap package in R (Figure 4 and Table S11) (Ihaka and Gentleman, 1996).

The available sequences of homologous genes involved in PE biosynthesis in Malpighiales (prenyltransferase, farnesyl diphosphate synthase, geranylgeranyl diphosphate synthase and casbene synthase; Costa *et al*., 2010) were obtained by searching the National Center for Biotechnology Information (NCBI) database. Jatropha protein sequences were mapped against the homologous genes by tblastn with an e‐value of 1e^‐10^, and genes with identity <80% and length coverage <80% were removed. The heatmap was constructed based on log_10_RPKM values (Figure 5b).

## Supporting information


**Figure S1** Schematic flowchart of assembly strategy.
**Figure S2** Insert size distributions of Illumina mate paired reads.
**Figure S3** Schematic of genetic map anchoring.
**Figure S4** Phylogeny tree using 42 orthologous genes based on synteny among eight species.
**Figure S5** Morphology of eight Jatropha species.
**Figure S6** GO enrichment (molecular functions) of DEGs between female and male flowers.
**Figure S7** Ks distribution of Jatropha and castor bean.
**Figure S8** Length distribution of Illumina zero depth blocks.
**Figure S9** GO classification of Jatropha genes.
**Table S1** Raw reads statistics of Pacbio and Illumina for *J. curcas* var. CN.
**Table S2** Marker information for genetic map construction.
**Table S3** Evaluation of the gene spacing completeness of Jatropha genome assembly.
**Table S4** Level of heterozygosity in *J. curcas* CN genome.
**Table S5** Statistics of RNA‐seq raw reads, *de novo* transcript assembly and transcript mapping.
**Table S6** Statistics of transcripts library for annotation.
**Table S7** Repeat annotation in the *Jatropha* genome assembly.
**Table S8** Differentially expressed transcription factors between female and male flowers.
**Table S9** Putative acyl lipid genes in Jatropha.
**Table S10** Differentially expressed putative acyl lipid genes in Jatropha.
**Table S11** The most significant GO terms of DEGs in early and late stages in putative acyl lipid biosynthesis.
**Table S12** RPKM values of putative casbene synthase in Jatropha.
**Table S13** Comparison of Jatropha genome assemblies.
**Table S14** Summary of zero depth block.
**Table S15** Ks values between the homologous genes at the physical cluster of diterpenoid biosynthesis genes of Jatropha and castor bean.
**Table S16** Frequency of five‐mers in zero depth blocks and non‐zero depth blocks.
**Table S17** SSR loci development from *J. curcas* CN.
**Table S18** Unit size of identified SSR loci.
**Data S1** Plant materials.
**Data S2** Genetic map construction and scaffold anchoring.
**Data S3** Transcriptome assembly and expression analysis.
