# Supplementary material for: Genome sequence of Jatropha curcas L., a non‐edible biodiesel plant, provides a resource to improve seed‐related traits
Source: Plant Biotechnol J. 2018 Sep 11;17(2):517–30. doi: 10.1111/pbi.12995 (PMC6335072; doi:10.1111/pbi.12995)
Supplement: Supplementary file 1 — Figure S1 Schematic flowchart of assembly strategy. Figure S2 Insert size distributions of Illumina mate paired reads. Figure S3 Schematic of genetic map anchoring. Figure S4 Phylogeny tree using 42 orthologous genes based on synteny among eight species. Figure S5 Morphology of eight Jatropha species. Figure S6 GO enrichment (molecular functions) of DEGs between female and male flowers. Figure S7 Ks distribution of Jatropha and castor bean. Figure S8 Length distribution of Illumina zero depth blocks. Figure S9 GO classification of Jatropha genes. Table S1 Raw reads statistics of Pacbio and Illumina for J. curcas var. CN. Table S2 Marker information for genetic map construction. Table S3 Evaluation of the gene spacing completeness of Jatropha genome assembly. Table S4 Level of heterozygosity in J. curcas CN genome. Table S5 Statistics of RNA‐seq raw reads, de novo transcript assembly and transcript mapping. Table S6 Statistics of transcripts library for annotation. Table S7 Repeat annotation in the Jatropha genome assembly. Table S8 Differentially expressed transcription factors between female and male flowers. Table S9 Putative acyl lipid genes in Jatropha. Table S10 Differentially expressed putative acyl lipid genes in Jatropha. Table S11 The most significant GO terms of DEGs in early and late stages in putative acyl lipid biosynthesis. Table S12 RPKM values of putative casbene synthase in Jatropha. Table S13 Comparison of Jatropha genome assemblies. Table S14 Summary of zero depth block. Table S15 Ks values between the homologous genes at the physical cluster of diterpenoid biosynthesis genes of Jatropha and castor bean. Table S16 Frequency of five‐mers in zero depth blocks and non‐zero depth blocks. Table S17 SSR loci development from J. curcas CN. Table S18 Unit size of identified SSR loci. Data S1 Plant materials. Data S2 Genetic map construction and scaffold anchoring. Data S3 Transcriptome assembly and expression analysis. [file PBI-17-517-s001.docx]

Data S1. Plant materials.

Genomic DNA was prepared from young *Jatropha curcas* CN leaves using the CTAB method (Allen *et al.*, 2006). RNA samples were prepared from the leaf tissue of nine *Jatropha* accessions, including *J. aconitifolia* (SRR5974854), *J. cinerea* (SRR5974851), *J. curcas* CN (SRR5974846), *J. curcas* M10 (SRR5974853), *J. gossypiifolia* (SRR5974852), *J. integerrima* (SRR5974859), *J. macrantha* (SRR5974860), *J. multifida* (SRR5974840), and *J. podagrica* (SRR5974839) (Supplementary Figure 5), as well as *Ricinus communis* (SRR5974838) and from stem (SRR5974856), root (SRR5974855), male flower (SRR5974843), and female flower (SRR5974844) tissue, as well as seed endosperm tissue from fruit at four different developmental stages (immature [SRR5974841], green [SRR5974842], yellow [SRR5974857], and brown fruit [SRR5974858]), of *J. curcas* CN, and sequenced on the Illumina HiSeq2000 platform (Supplementary Table 5).

Data S2. Genetic map construction and scaffold anchoring.

To construct a genetic map, 108 F_2_ lines derived from a cross between *J. curcas* CN and *J. curcas* M10 were genotyped using GBS (Supplementary Figure 3) (Elshire *et al.*, 2011). Genomic DNA was extracted from each line and fragmented using the restriction enzyme ApeKI. The quality and quantity of the PCR fragments were checked using a Bioanalyzer2100 (Agilent Technologies) before library construction. The sequencing reads produced by Illumina Hiseq2000 were mapped to our genome assembly using BWA v0.7.15 (Li and Durbin, 2009). Heterozygous genotypes were determined using SAMtools v1.3 (mapping quality ≥ 30, mapping depth ≥ 5, heterozygosity ≤ 10, missing < 10) (Li *et al.*, 2009). Using JoinMap4, 11 linkage groups were constructed using the regression-mapping algorithm with Kosambi mapping function. For scaffold anchoring, another genetic map was constructed with a different set of markers (mapping quality ≥ 30, mapping depth ≥ 3, heterozygosity ≤ 50, missing < 15). The two genetic maps were used to anchor scaffolds into 11 pseudochromosomes using ALLMAPS (Supplementary Table 2) (Tang *et al.*, 2015). Mapping depth was calculated from 7.8 Mbp of the reference assembly, where 90% or more of the 108 lines were mapped.

Data S3. Transcriptome assembly and expression analysis.

Approximately 4.1–5.5 Gbp of RNA-seq data were produced from 23 libraries on the Illumina Hiseq2000 platform, and the paired-end reads from each sample were *de novo* assembled using Trinity v2.2.0 with default parameters (Supplementary Table 5) (Grabherr *et al.*, 2011). CDS were predicted from transcripts using a Perl script, transcript_fasta_to_ORF_pics.pl, provided in the Trinity package. Redundant sequences were removed by CD-HIT (Li and Godzik, 2006). Complete protein sequences were compared among species and samples using an all-to-all approach with BLASTP (Camacho *et al.*, 2009). For expression analysis, reads per kilobase of transcript per millions of mapped reads (RPKM) values were calculated using GFOLD V1.1.4 by mapping RNA reads against Jatropha annotation data (Feng *et al.*, 2012). For heatmap analysis, log_10_(RPKM + 1) values were used for visualization. DEGs were defined using GFOLD diff program (GFOLD > 1 or GFOLD < -1) (Supplementary Table 8, 9, 10). GO enrichment of DEGs was analyzed using BiNGO (Maere *et al.*, 2005). The genes with RPKM value of 0.25 or lower were defined as unexpressed genes for tissue specific expression (Feng *et al.*, 2012). The Venn diagram was drawn using the Vennerable package in R (Ihaka and Gentleman, 1996).


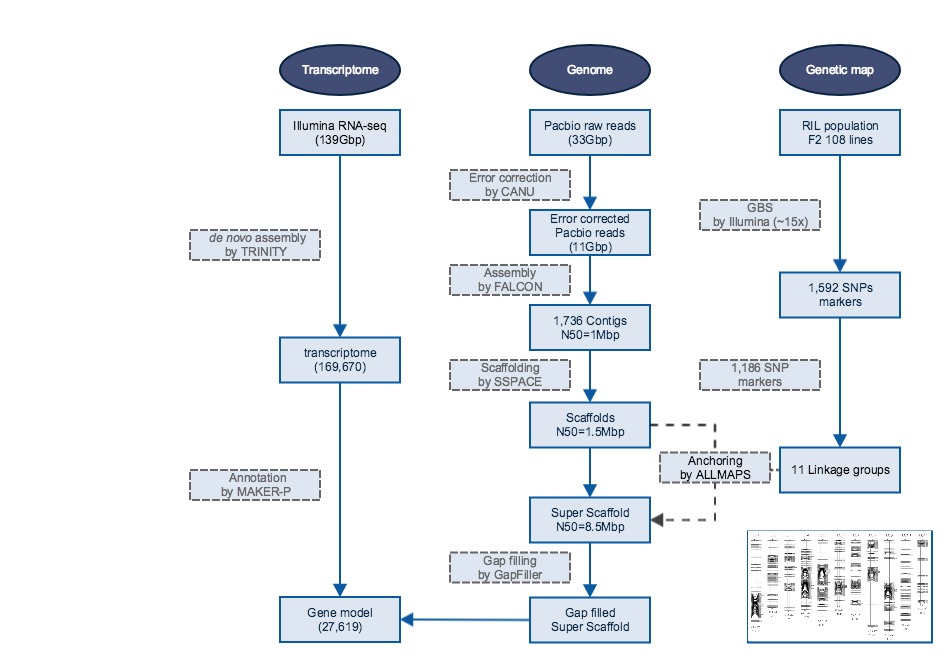


Supplementary Figure 1. Schematic flowchart of assembly strategy.

Contigs were assembled using Pacbio long reads after error correction. With three sets of Illumina mate pair reads, contigs were scaffolded, then anchored into pseudo chromosomes using genetic maps. The gaps between contigs in scaffolds and between scaffolds in superscaffolds were spanned using Illumina paired end reads (~48.5 Gbp). In total, 139 Gbp Illumina RNA-seq reads were *de-novo* assembled then clustered into 169,670 transcripts to construct library for gene annotation. A total of 27,619 gene models were annotated.


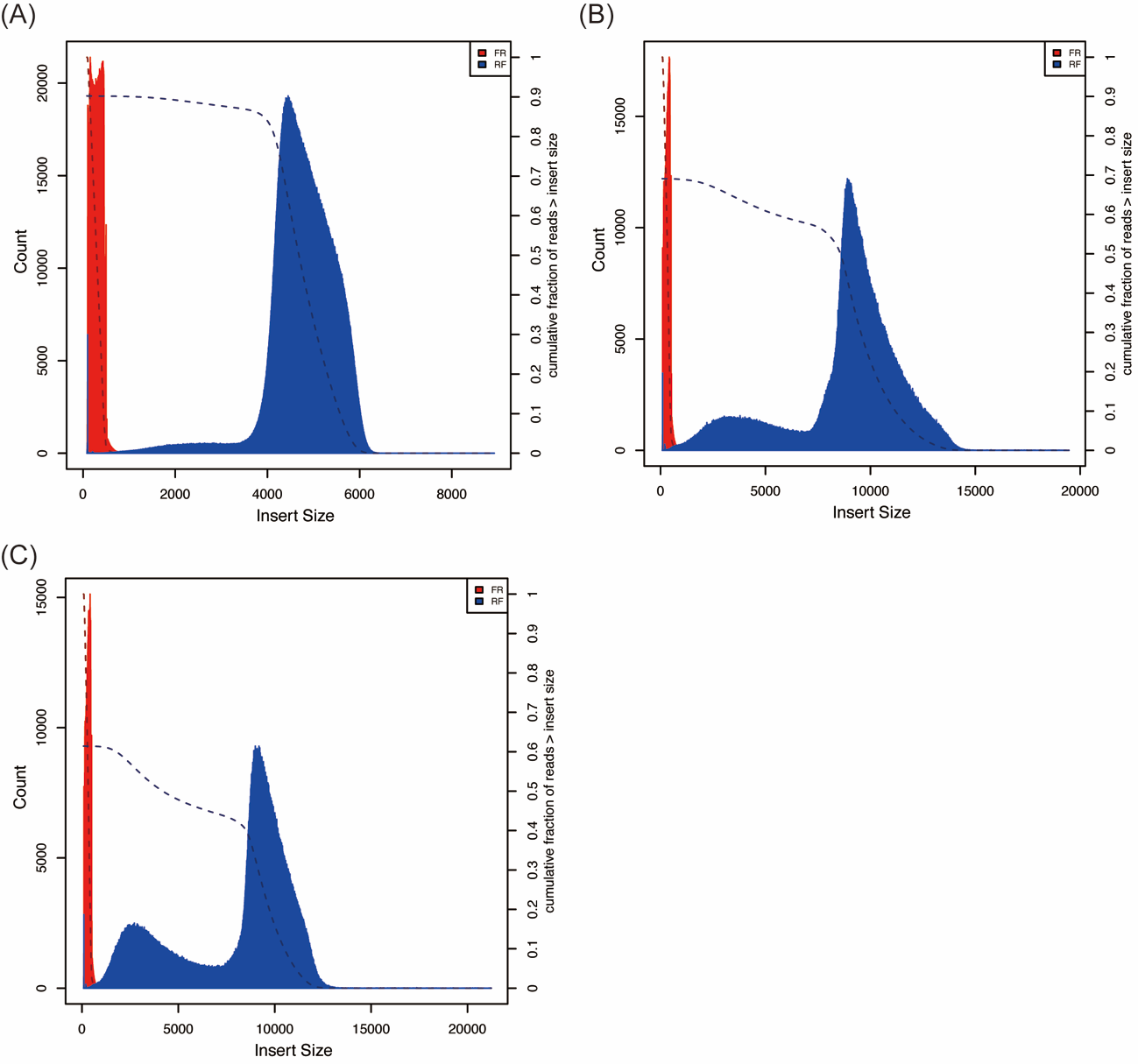


Supplementary Figure 2. Insert size distributions of Illumina mate paired reads.

Mate pair reads from three different libraries were mapped against contigs assembled using only Pacbio. (A) Distribution of distances between mate pairs from MP1 library (5 kbp). (B) Distribution of distances between mate pairs from MP2 library (10 kbp). (C) Distribution of distances between mate pairs from MP3 library (10 kbp).


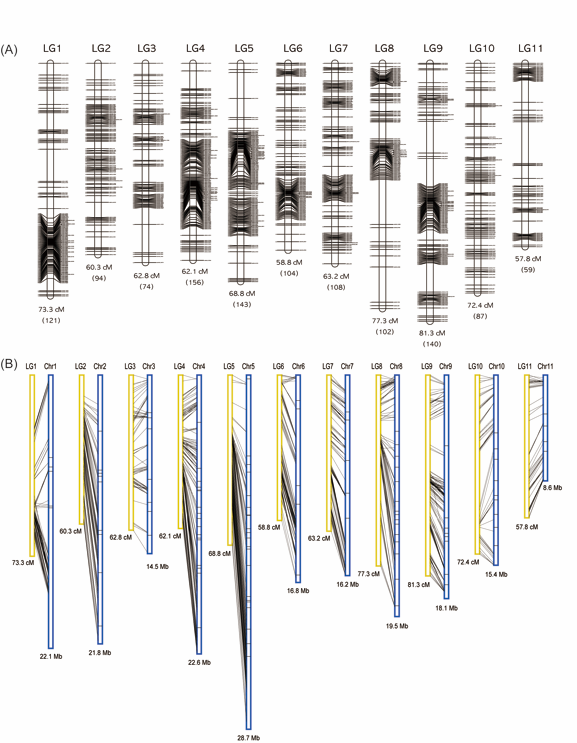


Supplementary Figure 3. Schematic of genetic map anchoring.

(A) Genetic map from F2 population derived from a cross between *J. curcas* CN and *J. curcas* M10. Genetic map, representing 738.1 cM, was constructed using 1,188 markers out of 1,592 markers identified. (B) Schematic of superscaffold anchoring. Out of 917 scaffolds, 116 scaffolds (blue) were anchored to 11 linkage groups (yellow) using 1,770 unique markers. Gaps in the superscaffolds are indicated by black lines in the blue boxes of superscaffolds.


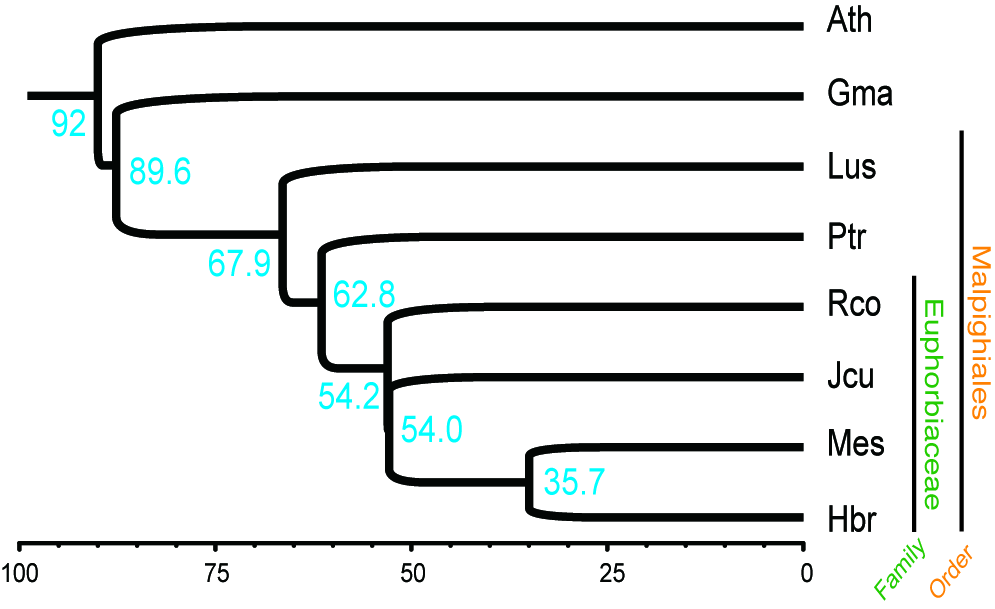


Supplementary Figure 4. Phylogeny tree using 42 orthologous genes based on synteny among eight species.

Four species in Euphorbiaceae family (*J. curcas*, *M. esculenta*, *H. brasilensis* and *R. communis*), two species in Malpighiales order (*P. trichocarpa* and *L. usitatissimum*) and two outgroups (*G. max* and *A. thaliana*) were included for the analysis. The tree was constructed by baysian method using BEAST with JTT+G as the best-fit model. The root divergence time was set to the estimated divergence time between Brassicales and Fabales (~92 mya).

Supplementary Figure 5. Morphology of eight Jatropha species.

Supplementary Figure 6. GO enrichment (molecular functions) of DEGs between female and male flowers.

The enrichment was analyzed by BiNGO.


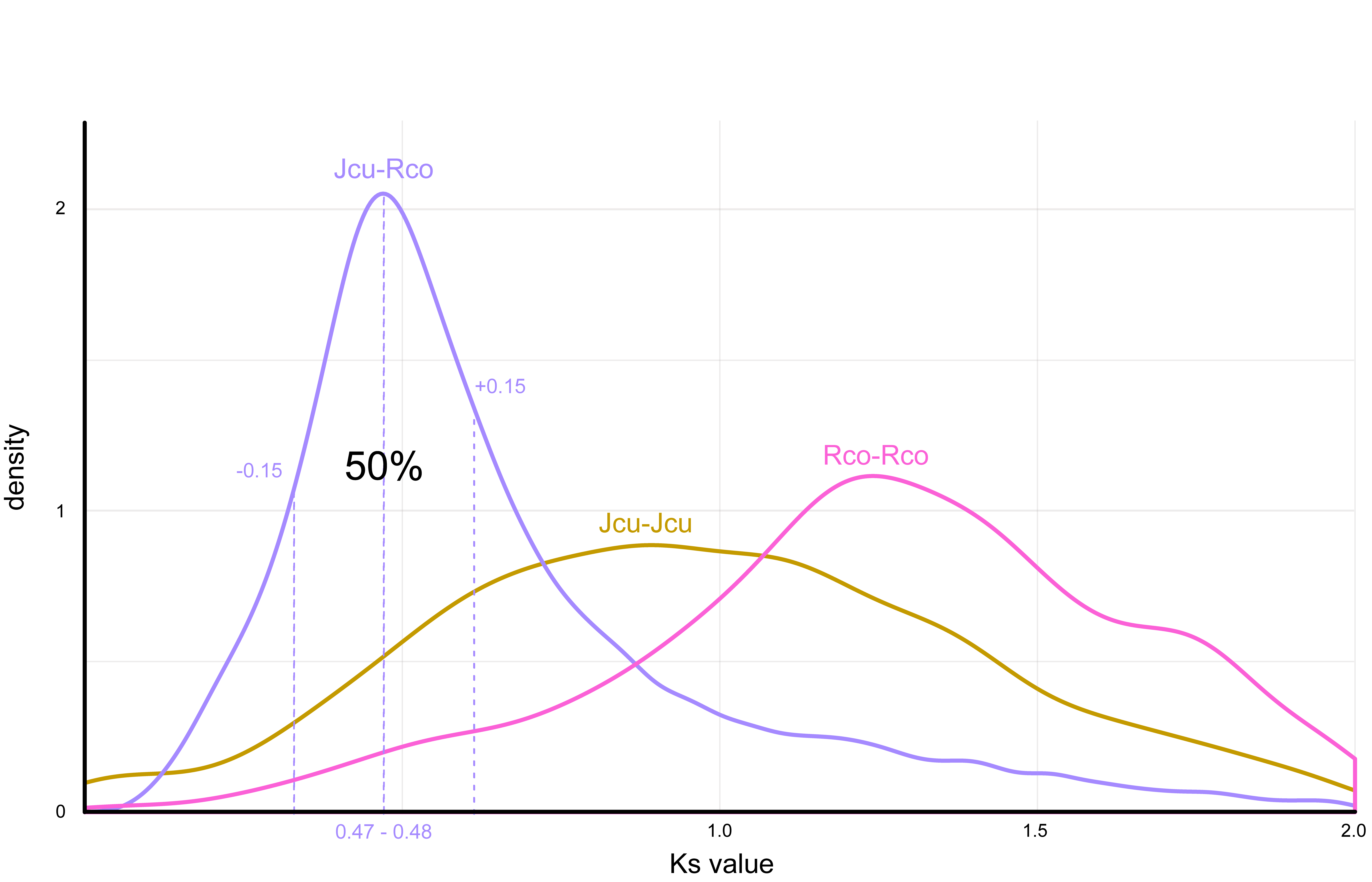


Supplementary Figure 7. Ks distribution of Jatropha and castor bean.

Ks distributions within Jatropha (Jcu-Jcu) and castor bean (Rco-Rco) are indicated by yellow and pink lines. Ks distribution between Jatropha and castor bean (Jcu-Rco) is indicated by purple lines. A bin of 0.47-0.48 has the highest density and 0.32 – 0.63 (±0.15 from the mode bin) contains 50% of total Ks distribution (Jcu: *J. curcas* and Rco: *Ricinus communis*).

Supplementary Figure 8. Length distribution of Illumina zero depth blocks.

X axis indicates the length of zero depth blocks. Y axis indicates the number of zero depth blocks in log2 values.


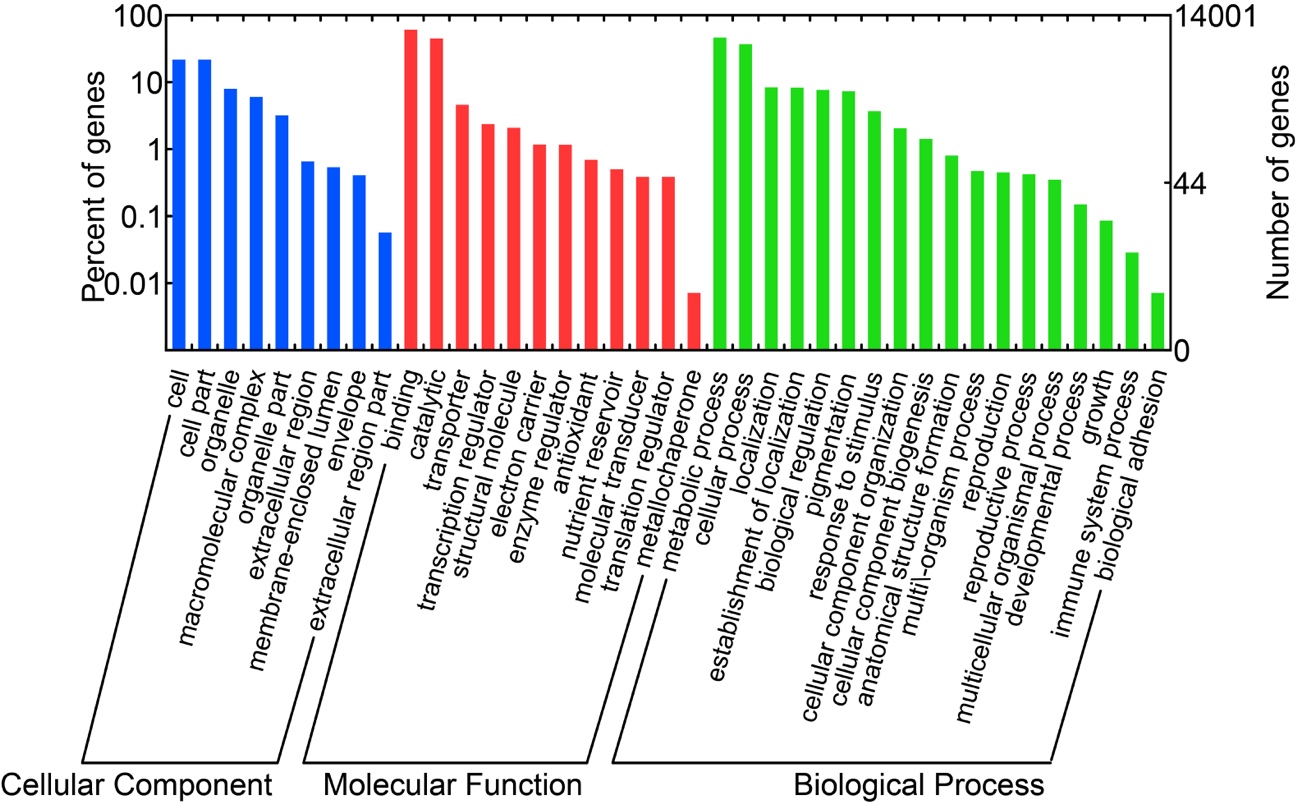


Supplementary Figure 9. GO classification of Jatropha genes.

The enrichment was visualized using WEGO (http://wego.genomics.org.cn/cgi-bin/wego/index.pl).

Supplementary Table 1. Raw reads statistics of Pacbio and Illumina for *J. curcas* var. CN.

PE, MP and PB indicates Illumina paired end read, Illumina mate pair read and Pacbio long read, respectively.

|  | PE | MP1 | MP2 | MP3 | PB | Error-corrected and trimmed PB |
| --- | --- | --- | --- | --- | --- | --- |
| library size | 200 | 5k | 10k | 10k | 20k | 20k |
| Average read length | 101 | 101 | 101 | 101 | 9,011 | 9,493 |
| # of reads | 480,151,696 | 481,318,814 | 462,296,542 | 378,035,866 | 3,616,402 | 1,153,108 |
| total bases | 48,495,321,296 | 48,613,200,214 | 46,691,950,742 | 38,181,622,466 | 32,586,481,078 | 10,946,920,825 |
| min read length |  |  |  |  | 500 | 1,000 |
| max read length |  |  |  |  | 49,575 | 39,073 |

Supplementary Table 2. Marker information for genetic map construction.

Two genetic maps were constructed for scaffold anchoring. GBS sequencing reads were mapped to scaffolds having 15x mapping depth on average. Two sets of genetic markers consisting of 1,186 and 864 markers were filtered using different criteria. When scaffolds had two or more markers, they were located and oriented while scaffolds with one marker were located but not oriented.

|  | genetic map 1 | genetic map 2 | Consensus map | | |
| --- | --- | --- | --- | --- | --- |
| Number of linkage groups | 11 | 11 | Anchored | Oriented | Unplaced |
| Number of unique markers | 1,186 | 864 | 1,770 | 1,713 | 17 |
| Number of markers per 1Mb | 6 | 5 | 9 | 10 | 0 |
| N50 scaffolds | 45 | 37 | 45 | 40 | 6 |
| scaffolds | 114 | 90 | 116 | 86 | 801 |
| scaffolds with 1 marker | 28 | 31 | 24 | - | 9 |
| scaffolds with 2 markers | 20 | 11 | 16 | 15 | 1 |
| scaffolds with 3 markers | 11 | 6 | 13 | 9 | - |
| scaffolds with >=4 markers | 55 | 42 | 63 | 62 | 1 |
| total bases | 205,388,765 | 161,312,253 | 204,156,632 | 181,104,912 | 135,182,730 |
| total bases (%) | 60.5 | 47.5 | 60.2 | 53.4 | 39.8 |

Supplementary Table 3. Evaluation of the gene spacing completeness of Jatropha genome assembly.

Both repeat masked and un-masked genome assemblies were used for CEGMA and BUSCO. The number and the percentage of completely and partially (in parentheses) mapped genes were counted.

|  | Number of ^a^CEGMA | Percentage of CEGMA |
| --- | --- | --- |
| Masked | 137 (165) | 55.2 (66.5) |
| Un-Masked | 213 (241) | 85.9 (97.2) |
|  | Number of ^b^BUSCO | Percentage of BUSCO |
| Masked | 1,150 (1,251) | 79.9 (86.9) |
| Un-Masked | 1,188 (1,265) | 82.5 (87.8) |

^a^CEGMA: Core eukaryotic genes mapping approach.

^b^BUSCO: Benchmarking universal single-copy orthologs.

Supplementary Table 4. Level of heterozygosity in *J. curcas* CN genome.

The number and the density per 1kbp of variants were searched in Jatropha genome. SNV indicates single-nucleotide variation and INDEL indicates insert and deletion.

|  | SNV | INDEL |
| --- | --- | --- |
| No. of Variants | 199,200 | 20,364 |
| Variants density (per 1kb) | 0.59 | 0.06 |
| No. of Variants in genes | 14,698 | 4,154 |
| Variants density in genes (per 1kb) | 0.18 | 0.05 |
| No. of Variants in exons | 6,115 | 841 |
| Variants density in exons (per 1kb) | 0.17 | 0.02 |
| No. of Variants in intergenic regions | 184,502 | 16,210 |
| Variants density in intergenic regions (per 1kb) | 0.72 | 0.06 |
| No. of Variants in repeat regions | 167,248 | 12,939 |
| Variants density in repeat regions (per 1kb) | 0.82 | 0.06 |

Supplementary Table 5. Statistics of RNA-seq raw reads, *de novo* transcript assembly and transcript mapping.

Transcripts were *de-novo* assembled in leaf, flower, endosperm, stem and root tissues of *J. curcas* var. CN and in leaf tissues of *J. curcas* var. M10, *J. aconitiforia*, *J. cineria*, *J. gossypifolia*, *J. intergerrima*, *J. macrantha*, *J. multifida*, *J. podagrica* and *R. communis* using Trinity v2.2.1. To assess the quality of the genome assembly, transcripts were mapped to the genome assembly.

| Tissue type | Read Count | Total Bases | GC (%) | Q20 (%) | Transcripts | Number of reads mapped | Percentage of mapped transcripts | Number of reads mapped >90% | Percentage of mapped transcripts (>90%) |
| --- | --- | --- | --- | --- | --- | --- | --- | --- | --- |
| Leaf | 54,483,884 | 5,502,872,284 | 46.82 | 95.70 | 76,409 | 75,479 | 98.78 | 71,829 | 94.01 |
| Stem | 40,867,354 | 4,127,602,754 | 45.79 | 95.94 | 48,926 | 48,146 | 98.41 | 45,373 | 92.74 |
| Root | 47,286,950 | 4,775,981,950 | 43.03 | 96.99 | 92,986 | 86,375 | 92.89 | 82,122 | 88.32 |
| Female flower 1 | 44,507,996 | 4,495,307,596 | 43.13 | 97.21 | 74,297 | 73,925 | 99.50 | 70,133 | 94.40 |
| Female flower 2 | 41,380,084 | 4,179,388,484 | 43.19 | 97.26 | 73,222 | 72,918 | 99.58 | 69,457 | 94.86 |
| Female flower 3 | 44,031,020 | 4,447,133,020 | 43.2 | 97.16 | 77,374 | 76,802 | 99.26 | 73,218 | 94.63 |
| Male flower 1 | 41,973,046 | 4,239,277,646 | 42.59 | 97.01 | 63,153 | 62,663 | 99.22 | 59,456 | 94.15 |
| Male flower 2 | 51,695,026 | 5,221,197,626 | 42.75 | 97.24 | 65,920 | 65,251 | 98.99 | 61,825 | 93.79 |
| Male flower 3 | 45,183,174 | 4,563,500,574 | 42.63 | 97.17 | 63,011 | 62,672 | 99.46 | 59,541 | 94.49 |
| Endosperm of immature fruit 1 | 50,698,068 | 5,120,504,868 | 42.99 | 97.20 | 43,857 | 43,743 | 99.74 | 42,260 | 96.36 |
| Endosperm of immature fruit 2 | 51,637,976 | 5,215,435,576 | 43.03 | 97.28 | 76,333 | 76,093 | 99.69 | 72,502 | 94.98 |
| Endosperm of green fruit 1 | 50,477,040 | 5,098,181,040 | 43.3 | 97.24 | 68,424 | 68,120 | 99.56 | 64,841 | 94.76 |
| Endosperm of green fruit 2 | 44,329,162 | 4,477,245,362 | 42.92 | 97.25 | 80,085 | 79,783 | 99.62 | 76,071 | 94.99 |
| Endosperm of green fruit 3 | 45,817,384 | 4,627,555,784 | 43.09 | 97.35 | 73,298 | 73,009 | 99.61 | 69,418 | 94.71 |
| Endosperm of Yellow fruit 1 | 46,267,516 | 4,673,019,116 | 43.87 | 97.15 | 27,601 | 27,534 | 99.76 | 26,833 | 97.22 |
| Endosperm of Yellow fruit 2 | 43,145,730 | 4,357,718,730 | 43.68 | 97.26 | 65,655 | 65,035 | 99.06 | 62,171 | 94.69 |
| Endosperm of Yellow fruit 3 | 51,849,048 | 5,236,753,848 | 43.6 | 97.67 | 66,491 | 66,185 | 99.54 | 63,207 | 95.06 |
| Endosperm of brown fruit 1 | 49,381,074 | 4,987,488,474 | 43.76 | 97.56 | 61,448 | 59,690 | 97.14 | 56,345 | 91.70 |
| Endosperm of brown fruit 2 | 54,120,978 | 5,466,218,778 | 43.74 | 97.63 | 68,572 | 67,601 | 98.58 | 64,354 | 93.85 |
| Endosperm of brown fruit 3 | 56,587,544 | 5,715,341,944 | 42.99 | 97.72 | 67,883 | 67,386 | 99.27 | 63,797 | 93.98 |
| *J. curcas* M10 | 40,398,958 | 4,080,294,758 | 44.44 | 97.27 | 56,894 |  |  |  |  |
| *J. aconitiforia* | 46,954,424 | 4,742,396,824 | 43.55 | 97.03 | 116,859 |  |  |  |  |
| *J. cineria* | 46,188,556 | 4,665,044,156 | 44.47 | 97.2 | 52,791 |  |  |  |  |
| *J. gossypifolia* | 46,985,974 | 4,745,583,374 | 43.97 | 97.31 | 68,874 |  |  |  |  |
| *J. intergerrima* | 48,935,062 | 4,942,441,262 | 43.41 | 97.43 | 66,446 |  |  |  |  |
| *J. macrantha* | 52,740,022 | 5,326,742,222 | 50.23 | 94.21 | 86,199 |  |  |  |  |
| *J. multifida* | 46,345,858 | 4,680,931,658 | 43.69 | 97.25 | 26,004 |  |  |  |  |
| *J. podagrica* | 43,174,134 | 4,360,587,534 | 43.95 | 97.03 | 70,200 |  |  |  |  |
| *Ricinus communis* | 52,892,602 | 5,342,152,802 | 43.43 | 97.09 | 76,716 |  |  |  |  |

Supplementary Table 6. Statistics of transcripts library for annotation.

All transcripts *de novo* assembled from five different tissues of *J. curcas* CN were clustered to remove redundant transcripts.

| Number of transcripts | 169,670 |
| --- | --- |
| Number of transcripts in N50 | 20,557 |
| GC Content of transcripts (%) | 39.58 |
| Minimum transcripts length | 150 |
| Maximum transcripts length | 15,609 |
| Mean transcripts length | 584.14 |
| Standard deviation of transcripts length | 810.92 |
| Median transcripts length | 255 |
| N50 transcripts length | 1,353 |

Supplementary Table 7. Repeat annotation in the *Jatropha* genome assembly.

Repetitive sequences are annotated on *Jatropha* genome assembly and Illumina zero depth blocks.

|  |  |  | Length occupied | Total repeat (%) | Genome (%) | Zero depth block (%) |
| --- | --- | --- | --- | --- | --- | --- |
| Retrotransposon | LTR | Gypsy | 96,907,111 | 48.10 | 28.54 | 41.44 |
|  |  | Copia | 27,090,101 | 13.44 | 7.98 | 12.50 |
|  |  | Caulimovirus | 5,596,124 | 2.78 | 1.65 | 5.75 |
|  |  | Cassandra | 289,321 | 0.14 | 0.09 | 0.07 |
|  |  | Others | 891,704 | 0.44 | 0.26 | 0.17 |
|  | LINE |  | 9,296,874 | 4.61 | 2.74 | 2.18 |
|  | SINE |  | 351,338 | 0.17 | 0.10 | 0.05 |
|  | Total |  | 140,422,573 | 69.69 | 41.36 | 62.17 |
| DNA transposon |  |  | 18,880,992 | 9.37 | 5.56 | 6.79 |
| Others |  |  | 42,185,467 | 20.94 | 12.43 | 31.04 |
| Total |  |  | 201,489,032 | 100 | 59.35 | 100 |

Supplementary Table 8. Differentially expressed transcription factors between female and male flowers.

| ^a^Transcription Factor | No. of genes | Female up-regulated | Male up-regulated |
| --- | --- | --- | --- |
| **AP2-EREBP** | **42** | **37** | 5 |
| bZIP | 6 | 3 | 3 |
| C2C2-GATA | 6 | 5 | 1 |
| CCAAT | 2 | 2 | 0 |
| Coactivator | 1 | 1 | 0 |
| E2F-DP | 1 | 1 | 0 |
| HB | 9 | 9 | 0 |
| HSF | 5 | 4 | 1 |
| **MADS** | **15** | 8 | **7** |
| SBP | 1 | 1 | 0 |
| **WRKY** | **11** | 5 | 6 |
| Total | 99 | 76 | 23 |

^a^Transcription Factor: the most enriched transcription factors are in bold.

Supplementary Table 9. Putative acyl lipid genes in Jatropha.

|  | No. of genes |
| --- | --- |
| Arabidopsis acyl lipid genes | 775 |
| Putative acyl lipid genes in Jatropha | 862 |
| *DEGs | 305 |

*DEG: Differentially expressed genes (GFOLD >1 or GFOLD < -1).

Supplementary Table 10. Differentially expressed putative acyl lipid genes in Jatropha.

The number of putative acyl lipid biosynthesis genes in Jatropha, oil palm, castor bean, soybean and sesame are compared.

| ^a^Acyl lipid sub-pathways | No. of putative acyl lipid genes in Jatropha | NO. of DEGs | percentage of DEGs | No. of putative acyl lipid genes | | | |
| --- | --- | --- | --- | --- | --- | --- | --- |
|  |  |  |  | Oil palm | Castor bean | GmaxW82 | Sesame |
| Cutin Synthesis & Transport 1 | 82 | 39 | 47.56 | 92 | 84 | 98 | 87 |
| Cutin Synthesis & Transport 2 | 5 | 3 | 60.00 | 5 | 5 | 5 | 5 |
| Eukaryotic Galactolipid & Sulfolipid Synthesis | 49 | 11 | 22.45 | 55 | 50 | 77 | 53 |
| Eukaryotic Phospholipid Synthesis & Editing | 83 | 18 | 21.69 | 119 | 82 | 138 | 99 |
| Fatty Acid Elongation, Desaturation & Export From Plastid | 21 | 9 | 42.86 | 25 | 24 | 27 | 24 |
| **Fatty Acid Elongation & Wax** Biosynthesis | **261** | 109 | 41.76 | **250** | **263** | **389** | **285** |
| Fatty Acid Synthesis | 89 | 26 | 29.21 | 105 | 103 | 124 | 101 |
| Lipid Trafficking | 23 | 3 | 13.04 | 25 | 20 | 28 | 22 |
| Mitochondrial Fatty Acid & Lipoic Acid Synthesis | 26 | 5 | 19.23 | 29 | 25 | 38 | 28 |
| Mitochondrial Lipopolysaccharide Synthesis | 34 | 10 | 29.41 | 32 | 36 | 52 | 37 |
| Mitochondrial Phospholipid Synthesis | 15 | 4 | 26.67 | 17 | 19 | 25 | 17 |
| Oxylipin Metabolism 1 | 89 | 43 | 48.31 | 115 | 94 | 156 | 98 |
| Oxylipin Metabolism 2 | 71 | 30 | 42.25 | 90 | 76 | 124 | 75 |
| **Phospholipid Signaling** | **120** | 34 | 28.33 | **147** | **118** | **218** | **136** |
| Prokaryotic Galactolipid, Sulfolipid, & Phospholipid Synthesis 1 | 61 | 18 | 29.51 | 62 | 61 | 86 | 66 |
| Prokaryotic Galactolipid, Sulfolipid, & Phospholipid Synthesis 2 | 26 | 7 | 26.92 | 32 | 29 | 40 | 33 |
| Sphingolipid Biosynthesis 1 | 30 | 6 | 20.00 | 46 | 33 | 56 | 41 |
| Sphingolipid Biosynthesis 2 | 16 | 3 | 18.75 | 21 | 18 | 32 | 21 |
| Suberin Synthesis & Transport 1 | 81 | 43 | 53.09 | 86 | 80 | 114 | 96 |
| Suberin Synthesis & Transport 2 | 18 | 8 | 44.44 | 17 | 19 | 20 | 20 |
| Suberin Synthesis & Transport 3 | 21 | 8 | 38.10 | 21 | 22 | 28 | 24 |
| **Triacylglycerol Biosynthesis** | **114** | 46 | 40.35 | **130** | **111** | **164** | **119** |
| Triacylglycerol & Fatty Acid Degradation | 86 | 37 | 43.02 | 104 | 96 | 133 | 93 |
| Unknown | 76 | 24 | 31.58 | 74 | 77 | 114 | 84 |

^a^Acyl lipid sub-pathways: The most enriched five sub-pathways in Jatropha were in bold.

Supplementary Table 11. The most significant GO terms of DEGs in early and late stages in putative acyl lipid biosynthesis.

| ^a^Cluster | Biological Process | P-value | Molecular Function | P-value | Cellular Function | P-value |
| --- | --- | --- | --- | --- | --- | --- |
| Early  stage | phosphoinositide dephosphorylation | 7.07E-09 | phosphoric diester hydrolase activity | 2.10E-09 |  |  |
|  | lipid transport | 7.49E-04 | ATPase activity | 4.34E-04 |  |  |
|  | phosphatidylinositol metabolic process | 7.49E-04 | phosphatidylinositol phosphate kinase activity | 7.49E-04 |  |  |
|  | lipid biosynthetic process | 1.99E-03 | FMN binding | 1.25E-03 |  |  |
|  |  |  | transferase activity, transferring acyll groups, acyl groups converted into alkyl on transfer | 5.99E-03 |  |  |
|  |  |  | oxidoreductase activity | 6.33E-03 |  |  |
|  |  |  | lipid binding | 1.75E-02 |  |  |
|  |  |  | methyltransferase activity | 2.03E-02 |  |  |
|  |  |  | phosphoric diester hydrolase activity | 2.10E-09 |  |  |
| Late  stage | lipid transport | 1.31E-08 | lipid binding | 1.31E-08 | monolayer-surrounded lipid storage body | 6.68E-04 |
|  | multicellular organismal development | 2.81E-02 | oxidoreductase activity | 2.26E-04 |  |  |
|  | fatty acid biosynthesis process | 4.16E-02 | O-acyltransferase activity | 1.40E-02 |  |  |
|  |  |  | hydrolase activity, acting on ester bonds | 1.40E-02 |  |  |
|  |  |  | transcription factor activity | 4.16E-02 |  |  |

^a^Cluster: early stage indicates immature and green fruit; late stage indicates yellow and brown fruit.

Supplementary Table 12. RPKM values of putative casbene synthase in Jatropha.

IF: immature fruit, GF: green fruit, YF: yellow fruit, BF: brown fruit.

|  | IF | GF | YF | BF | Annotation |
| --- | --- | --- | --- | --- | --- |
| Jatcu.03g001402 | 0.0332 | 1.9227 | 13.6604 | 52.5288 | Jatropha curcas casbene synthase, chloroplastic-like (LOC105629811), mRNA |
| Jatcu.03g001403 | 0 | 0 | 0.0181 | 0 | PREDICTED: Jatropha curcas casbene synthase, chloroplastic (LOC105629821), mRNA |
| Jatcu.03g001404 | 0.0408 | 0.9203 | 7.8762 | 31.5334 | PREDICTED: Jatropha curcas casbene synthase, chloroplastic (LOC105629810), mRNA |
| Jatcu.03g001406 | 0 | 0 | 0 | 0 | Jatropha curcas casbene synthase, chloroplastic-like (LOC105629811), mRNA |
| Jatcu.05g000943 | 0 | 0 | 0.0516 | 0 | PREDICTED: Jatropha curcas casbene synthase, chloroplastic (LOC105645476), mRNA |
| Jatcu.08g001036 | 0 | 0 | 0 | 0 | Jatropha curcas casbene synthase (CASD168) mRNA, partial cds |
| Jatcu.U001471 | 0.0216 | 0.5342 | 5.7004 | 2.4088 | Jatropha curcas casbene synthase mRNA, complete cds |
| Jatcu.U001474 | 0.0302 | 0.0770 | 11.1247 | 6.7139 | Jatropha curcas casbene synthase (CASD168) mRNA, partial cds |
| Jatcu.U001480 | 0 | 0.0233 | 27.5176 | 12.1493 | Jatropha curcas casbene synthase mRNA, complete cds |
| Jatcu.U004706 | 0 | 0.0867 | 0 | 0 | PREDICTED: Jatropha curcas casbene synthase, chloroplastic (LOC105645476), mRNA |

Supplementary Table 13. Comparison of Jatropha genome assemblies.

|  | ^a^Hirakawa et al. 2012 | ^b^Wu et al. 2015 | | Illumina-only assembly | | Pacbio assembly | | |
| --- | --- | --- | --- | --- | --- | --- | --- | --- |
|  | supercontig | contig | scaffold | contig | scaffold | contig | scaffold | superscaffold |
| Total length | 298M | 264M | 321M | 271M | 319M | 338M | 339M | 340M |
| Total number | 39,277 | 72,474  (>100bp) | 23,125  (>100bp) | 25,312 | 3,710 | 1,736 | 917 | 812 |
| N50 | 16k | 27k | 746k | 28k | 356k | 1,014k | 1,476k | 15,395k |
| Number of genes annotated | 30,203 |  | 27,172 |  | 21,957 |  |  | 27,619 |
| Assembly programs | ^c^SOAPdenovo  (kmer 31)  ^d^PCAP.rep | ^c^SOAPdenovo  (kmer 35) | | ^e^ALLPATH-LG | | ^f^Falcon | ^g^SSAPCE | ^h^ALLMAPS |
| Sequencing platform | Sanger method  Roche/454  Illumina-solexa GAII | Illumina GAII  Illumina Hiseq | | Illumina Hiseq | | Pacbio  Illumina Hiseq | | |

^a^Hirakawa, H. *et al.* Upgraded genomic information of Jatropha curcas L. *Plant Biotechnol.* **29**(2), 123-130 (2012).

^b^Wu, P. *et al.* Integrated genome sequence and linkage map of physic nut (Jatropha curcas L.), a biodiesel plant. *Plant J.* **81,** 810–821 (2015).

^c^SOAPdenovo: Luo, R. *et al*. SOAPdenovo2: an empirically improved memory-efficient short-read de novo assembler. *GigaScience* **1,**18 (2012)

^d^PCAP.rep: Huang, X. *et al*. Application of a superword array in genome assembly. *Nucleic Acids Res* **34**, 201-5 (2006)

^e^ALLPATH-LG: Gnerre S. *et al*. High-quality draft assemblies of mammalian genomes from massively parallel sequence data. *PNAS* **108**(4),1513-1518 (2011)

^f^Falcon: Chin, CS. *et al*. Phased diploid genome assembly with single-molecule real-time sequencing. *Nat. Methods* **13**, 1050-1054 (2016)

^g^SSAPCE: Boetzer, M. *et al*. Scaffolding pre-assembled contigs using SSPACE. *Bioinforma.* *Oxf. Engl.* **27**, 578–579 (2011)

^h^ALLMAPS: Tang H. *et al*. ALLMAPS: robust scaffold ordering based on multiple maps. *Genome Biol*. **16**, 3 (2015)

Supplementary Table 14. Summary of zero depth block.

Illumina paired end reads were mapped against repeat masked genome sequence assembled using PacBio long reads.

| No. of Zero depth block without N | 48,162 |
| --- | --- |
| Total length of Zero depth block without N | 3,154,711 |
| No. of Zero depth block containing no repeats | 7,438 |
| Total length of Zero depth block containing no repeats | 104,590 |
| No. of Zero depth block containing repeats | 40,724 |
| Total length of Zero depth block containing repeats | 3,050,121 |

Supplementary Table 15. Ks values between the homologous genes at the physical cluster of diterpenoid biosynthesis genes of Jatropha and castor bean.

| ^a^Jatropha genes | ^b^Castor bean genes | ^c^Ks value |
| --- | --- | --- |
| Jatcu.03g001382.1 | 30169.m006275 | 1.06 |
| Jatcu.03g001383.1 | 30169.m006276 | 0.47 |
| Jatcu.03g001384.1 | 30169.m006275 | 0.90 |
| Jatcu.03g001385.1 | 30169.m006275 | 0.97 |
| Jatcu.03g001392.1 | 30169.m006275 | 0.74 |
| Jatcu.03g001393.1 | 30169.m006275 | 0.66 |
| Jatcu.03g001394.1 | 30169.m006275 | 1.09 |
| Jatcu.03g001396.1 | 30169.m006275 | 0.49 |
| Jatcu.03g001397.1 | 30169.m006275 | 0.91 |
| Jatcu.03g001399.1 | 30169.m006285 | 0.76 |
| Jatcu.03g001400.1 | 30169.m006275 | 0.98 |
| Jatcu.03g001402.1 | 30169.m006283 | 0.99 |
| Jatcu.03g001403.1 | 30169.m006283 | 0.63 |
| Jatcu.03g001404.1 | 30169.m006283 | 0.93 |
| Jatcu.03g001405.1 | 30169.m006285 | 0.53 |
| Jatcu.03g001406.1 | 30169.m006286 | -2.00 |
| Jatcu.03g001408.1 | 30169.m006276 | 0.60 |

^a^Jatropha genes: the genes annotated as casbene synthase are in red.

^b^Castor bean genes: the genes functionally characterized as casbene synthase are in red.

^c^Ks value: the Ks value between 0.32 – 0.63 are in blue.

Supplementary Table 16. Frequency of five-mers in zero depth blocks and non-zero depth blocks.

The most frequent 20 five-mers in forward direction are included. AT-rich sequences are dominant both on zero depth and non-zero depth.

|  | 5-mers | Zero depth block | 5-mers | Non-zero depth block |
| --- | --- | --- | --- | --- |
| 1 | TTTTT | 1,880,593 | AAAAA | 18,202 |
| 2 | AAAAA | 1,878,671 | TTTTT | 17,342 |
| 3 | ATTTT | 1,301,903 | AAAAT | 15,334 |
| 4 | AAAAT | 1,298,863 | ATTTT | 15,206 |
| 5 | AATTT | 1,162,080 | TATAT | 13,634 |
| 6 | AAATT | 1,160,274 | ATATA | 13,568 |
| 7 | TTTTA | 1,005,977 | AAATT | 12,851 |
| 8 | TAAAA | 1,003,900 | AATTT | 12,723 |
| 9 | TATTT | 994,736 | TTTTA | 11,120 |
| 10 | AAATA | 991,468 | TAAAA | 11,115 |
| 11 | TTATT | 952,706 | AATAA | 10,920 |
| 12 | AATAA | 950,920 | TTATT | 10,693 |
| 13 | TTTAT | 915,961 | ATTAA | 10,282 |
| 14 | ATAAA | 913,329 | AATTA | 10,269 |
| 15 | TAATT | 906,069 | TTAAT | 10,237 |
| 16 | AATTA | 905,936 | AAATA | 10,229 |
| 17 | TTTAA | 861,800 | ATAAA | 10,214 |
| 18 | TTAAA | 859,509 | TAATT | 10,178 |
| 19 | TATAT | 830,539 | TTTAT | 10,156 |
| 20 | ATATA | 830,009 | TATTT | 10,080 |

Supplementary Table 17. SSR loci development from *J. curcas* CN.

| Total number of sequences examined | 812 |
| --- | --- |
| Total size of examined sequences(bp) | 339,501,388 |
| Total number of identified SSRs | 83,876 |
| Number of SSR containing sequences | 608 |
| Number of sequences containing more than 1 SSR | 537 |

Supplementary Table 18. Unit size of identified SSR loci.

| Unit size | Number of SSRs |
| --- | --- |
| 2 | 64,103 |
| 3 | 14,197 |
| 4 | 2,634 |
| 5 | 466 |
| 6 | 235 |
| 7 | 226 |
| 8 | 8 |
| 9 | 6 |
| 10 | 1 |
